# Experimental demonstration of an on-chip p-bit core based on stochastic magnetic tunnel junctions and 2D MoS_2_ transistors

**DOI:** 10.1038/s41467-024-48152-0

**Published:** 2024-05-15

**Authors:** John Daniel, Zheng Sun, Xuejian Zhang, Yuanqiu Tan, Neil Dilley, Zhihong Chen, Joerg Appenzeller

**Affiliations:** 1 Birck Nanotechnology Center, Purdue University, West Lafayette, IN 47907 USA; 2 Department of Physics and Astronomy, Purdue University, West Lafayette, IN 47907 USA; 3 School of Electrical and Computer Engineering, Purdue University, West Lafayette, IN 47907 USA

**Keywords:** Electronic devices, Magnetic devices

## Abstract

Probabilistic computing is a computing scheme that offers a more efficient approach than conventional complementary metal-oxide–semiconductor (CMOS)-based logic in a variety of applications ranging from optimization to Bayesian inference, and invertible Boolean logic. The probabilistic bit (or p-bit, the base unit of probabilistic computing) is a naturally fluctuating entity that requires tunable stochasticity; by coupling low-barrier stochastic magnetic tunnel junctions (MTJs) with a transistor circuit, a compact implementation is achieved. In this work, by combining stochastic MTJs with 2D-MoS_2_ field-effect transistors (FETs), we demonstrate an on-chip realization of a p-bit building block displaying voltage-controllable stochasticity. Supported by circuit simulations, we analyze the three transistor-one magnetic tunnel junction (3T-1MTJ) p-bit design, evaluating how the characteristics of each component influence the overall p-bit output. While the current approach has not reached the level of maturity required to compete with CMOS-compatible MTJ technology, the design rules presented in this work are valuable for future experimental implementations of scaled on-chip p-bit networks with reduced footprint.

## Abstract (web summary)

Probabilistic bits (p-bits) are the base units of probabilistic computing, a computing scheme offering a more efficient approach than conventional binary logic in various applications. Here, the authors report the realization of a p-bit core device by combining stochastic magnetic tunnel junctions and 2D MoS_2_ transistors on the same chip.

## Introduction

Computing is at a crossroads: just as the transistor-scaling driven by Moore’s Law has afforded improvements in conventional complementary metal-oxide–semiconductor (CMOS)-based computing performance, there is an inevitable slowing down due to fundamental device limits^1^. Furthermore, the inherently deterministic nature of conventional computing makes the current CMOS model unsuitable for contending with the continued future growth of applications such as in neuromorphic computing and Artificial Intelligence (AI)^2^.

A superior approach is that of probabilistic computing. In probabilistic computing, the key component is the probabilistic bit (or p-bit), a unit that fluctuates randomly, but controllably, between 0 and 1^3^. Indeed, a network of such p-bits can leverage their stochastic nature to function as efficient hardware accelerators for solving complex problems that are themselves inherently probabilistic. These problems, which lie at the core of many real-world machine learning applications and algorithms of AI, range in nature from combinatorial optimization problems (such as integer factorization) to recognition and classification^4–16^.

At its core, a p-bit requires a tunable stochastic element. While it should be noted that this can be implemented with standard CMOS technology^17–19^ and a significant device overhead, the resulting p-bit suffers from a large areal and energy footprint, as well as not offering true randomness^20^.

An ultra-compact approach for tunable randomness that yields the desired sigmoidal-shaped input/output characteristics, which is scalable and energy-efficient, is achieved by exploiting the physics of low-barrier fluctuating nanomagnets when coupled with existing magnetic tunnel junction (MTJ) technology. Such p-bit implementations using stochastic MTJs have been shown^21–25^, but as yet, the proof-of-concept implementations used alternate designs to the 3T-1MTJ p-bit structure that made it necessary to employ field-programmable gate arrays (FPGAs) or external circuitry, with orders of magnitude more transistors involved than required in the p-bit design explored in this work.

**Fig. 1:**
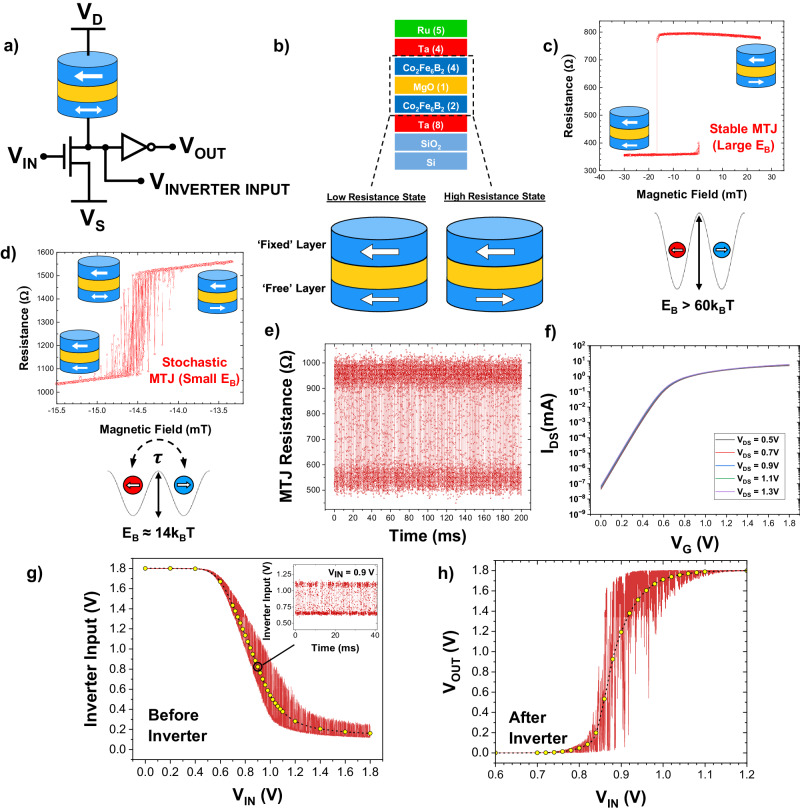
Implementing probabilistic bits (p-bits) with stochastic magnetic tunnel junctions (MTJs). **a** Schematic of the proposed p-bit design, comprised of a stochastic MTJ (magnetic moments represented by the white arrows) connected to the drain of an n-type transistor, forming the stochastic core of the p-bit. The current through the MTJ-transistor pair is determined by the drain (*V*_D_) and source (*V*_S_) voltages, while the input tunability comes from varying the gate voltage of the transistor (*V*_IN_). The inverter is used for thresholding and amplifying the voltage fluctuations between the MTJ and transistor (at *V*_INVERTER INPUT_). **b** Cross-section schematic of a typical MTJ stack, layer thicknesses in nm, and an explanation of the tunnel magnetoresistance (TMR) effect. **c** Minor loop of a stable MTJ, showing how the resistance changes deterministically as a function of magnetic field for a large energy barrier (*E*_B_) free layer (denoted by the representative double-well model where the potential barrier is much larger than ambient thermal energy, k_B_T). **d** Minor loop of a stochastic MTJ, showing how the resistance fluctuates between the parallel- and antiparallel state, with a mean dwell time *τ*, for a small energy barrier-free layer. **e** Example time-series resistance data for the fluctuating MTJ and **f** transfer characteristics of the transistor used to obtain the example p-bit’s output. **g** Graph showing the typical p-bit signal before the inverter’s operation, as a function of the transistor gate voltage (defined as *V*_IN_). The average at each point is shown by the dotted line while the inset figure shows example time-series data of the voltage fluctuations at the inverter’s input for *V*_IN_ = 0.9 V. **h** Graph showing the typical output of the full p-bit, *V*_OUT_, as a function of the input voltage, *V*_IN_. The time-averaged signal at each input voltage is represented by the dotted line.

In this work, an on-chip demonstration of the core of a p-bit, exhibiting tunable stochasticity, is reported. Using a variation of the 3T-1MTJ design proposed by Camsari et al.^26^, a stochastic MTJ is integrated with a high-performance MoS_2_ transistor next to each other on the same chip, experimentally showing the desired gate-controlled fluctuations at room temperature. Moreover, this article elucidates the impact and interaction of the various critical device characteristics shown in Fig. 1a, including that of the (i) MTJ, (ii) the transistor that is part of the p-bit core, and (iii) the inverter (see Fig. 1a). It is found that—against common wisdom—a large tunnel magnetoresistance (TMR) is not the best choice for p-bits; bimodal telegraphic fluctuations are highly undesirable and are a sign of a slow device; matching of the MTJ resistance and the transistor characteristics is crucial; and an ideal inverter with a large gain is incompatible with the desired p-bit operation.

## Results

### Implementing probabilistic bits (p-bits) with stochastic MTJs

At its core, a magnetic tunnel junction (MTJ) consists of two ferromagnetic layers separated by an ultrathin insulating layer (Fig. 1b). The “fixed” layer, which has the stronger magnetic moment, is used as the reference for the “free” layer, whose magnetic moment is more susceptible to being switched. Important MTJ parameters are tunnel magnetoresistance (TMR), which describes the difference in resistance between the parallel (P) and antiparallel (AP) arrangement of the two magnetic layers, and the energy barrier of the free layer, *E*_B,_ which needs to be overcome to toggle between the two resistance states^27–29^.

For stable MTJs, such as those used in spin-transfer torque magnetic random access memory (STT-MRAM) applications^30^, energy barriers are large and when the resistance is measured as an external magnetic field is swept, the resulting minor loop exhibits deterministic switching of the free layer. Figure 1c shows an example minor loop of a fabricated MTJ that was observed to be stable.

If this energy barrier is made smaller, through material changes or shape scaling^31^, the ambient thermal energy may be sufficient for the free layer to switch stochastically between the two resistance states (Fig. 1d). When biased at the center of this window, the signal is shown to be a naturally fluctuating output whereby the time spent in each resistance state (known as the dwell time, *τ*) may be described by the equation:

1 $$\tau={\tau }_{0}{e}\,^{{E}_{B}/{K}_{B}T},$$

where ${k}_{B}$ is the Boltzmann constant, $T$ is the temperature and ${\tau }_{0}$ is the “attempt time”, a material-dependent constant that is ~1 ns^32^. For in-plane stochastic MTJs, dwell times down to ~5 ns have been demonstrated^33,34^.

For p-bit applications, this source of natural stochasticity is ideal; by coupling a stochastic MTJ with an access transistor, and including an inverter for amplification, a compact voltage-controlled p-bit design is achieved (Fig. 1a)^26^.

The theoretical output from such a p-bit implementation, generated using modified experimental data from stochastic MTJs (Fig. 1e) and circuit simulations of transistor behavior (Fig. 1f), is shown before (Fig. 1g) and after (Fig. 1h) the inverter’s amplification. (For more details regarding the use of experimental data in the circuit simulations, please see Supplementary Note 1). The core of the p-bit, which includes the stochastic MTJ and the N-channel metal-oxide-semiconductor (NMOS) transistor, provides the tunable stochasticity while the inverter provides the thresholding and amplification of the stochastic signal. The resulting sigmoidal output allows for pinning at low- and high-input voltages while exhibiting the desired output fluctuations in the transition region.

The tunability in the output is controlled by varying the transistor gate voltage (*V*_IN_), where changes in the relative resistance of the transistor to the MTJ change the voltage at the inverter’s input. This voltage is then amplified through the inverter’s operation, allowing the output to be pinned to output-low for low *V*_IN_, and to output-high for high *V*_IN_. In the middle region, the stochastic resistance fluctuations from the MTJ manifest as tunable random voltage fluctuations in the p-bit output.

This design is discussed further in the following section, which shows the experimental realization of the p-bit core using a stochastic MTJ and a 2D-MoS_2_ transistor.

### Experimental demonstration of an on-chip p-bit core

For this demonstration, MTJ devices were first fabricated before those devices possessing sufficient TMR for a large read-signal were interconnected with appropriate resistance-matched field-effect transistor (FET) devices in a 1T-1MTJ configuration. It is desirable to have the transistor chosen such that the on-state FET resistance is at least two orders of magnitude smaller than the MTJ’s low-resistance state, *R*_P_, and that the off-state FET resistance is two orders of magnitude larger than the MTJ’s high-resistance state, *R*_AP_, to attain the maximum swing in the output voltage.

**Fig. 2:**
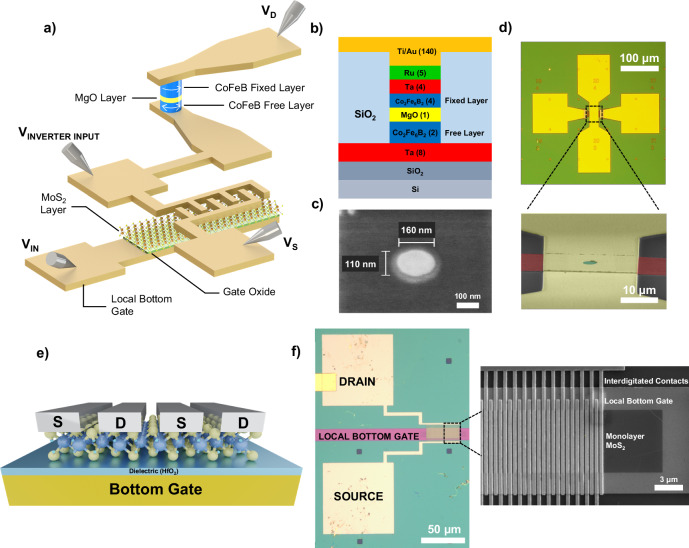
Fabricating an on-chip p-bit. **a** 3D schematic of the proposed design for an on-chip p-bit core, using a stochastic MTJ and 2D MoS_2_ field-effect transistor (FET). **b** Side cross-section view of the MTJ stack, with the fixed- and free-layer denoted. **c** Top scanning electron microscopy (SEM) view of an example MTJ pillar of the same nominal dimensions as the stochastic MTJ in the interconnected on-chip device. **d** Optical microscope and (false-color) tilted-SEM images of an example finished MTJ device. **e** Cross-section schematic of the 2D MoS_2_ FET. **f** Optical microscope and SEM images of the 2D FET, showing the interdigitated contacts that are used to attain high-current drives.

Figure 2a shows a schematic of the 1T-1MTJ configuration for the on-chip p-bit core. The detailed stack structure for the MTJs used in this demonstration is shown in Fig. 2b. The magnetic layer (CoFeB) thicknesses, were chosen to best yield MTJs with in-plane anisotropy due to two reasons: MTJs with in-plane anisotropies have been shown to be more resistant to spin-transfer torque (STT)-pinning^35^, and have also shown to fluctuate with time scales that are orders of magnitude faster than perpendicular-anisotropy MTJs^32,34,36^.

Figure 2c shows an SEM image of an example elliptical nanopillar with the same dimensions as the MTJs used in this demonstration, while Fig. 2d shows an optical microscope image of a finished MTJ device, along with a tilted-angle false-color SEM image of the MTJ region.

The interdigitated (IDT) monolayer (ML) MoS_2_ FETs are then fabricated alongside the completed MTJ devices. The cross-section of the FET is shown in Fig. 2e while an SEM image of a fabricated IDT ML MoS_2_ FET is shown in Fig. 2f, where a single IDT FET includes 20 sets of source/drain contacts, with *L*_ch_ ~ 150 nm and *W*_ch_ ~ 6.5 μm, for a total effective channel width of 130μm. ML MoS_2_ is chosen as the channel material of the drive transistor due to the low thermal budget fabrication process (to help preserve the performance of the fabricated stochastic MTJs, which suffer shorting in the SiO_2_ isolation layer for temperatures above ~400 °C), low contact resistance^37^, the large bandgap (1.8 eV), the high on-state performance of scaled 2D-MoS_2_ FETs^38^ and good electrostatic control achievable with ML MoS_2_. Although it would require significant experimental effort, it should be noted that the ultimate p-bit implementation would involve integrating advanced CMOS circuitry with unstable MTJs (rather than using MTJs in an MRAM array structure as nonvolatile memory elements).

**Fig. 3:**
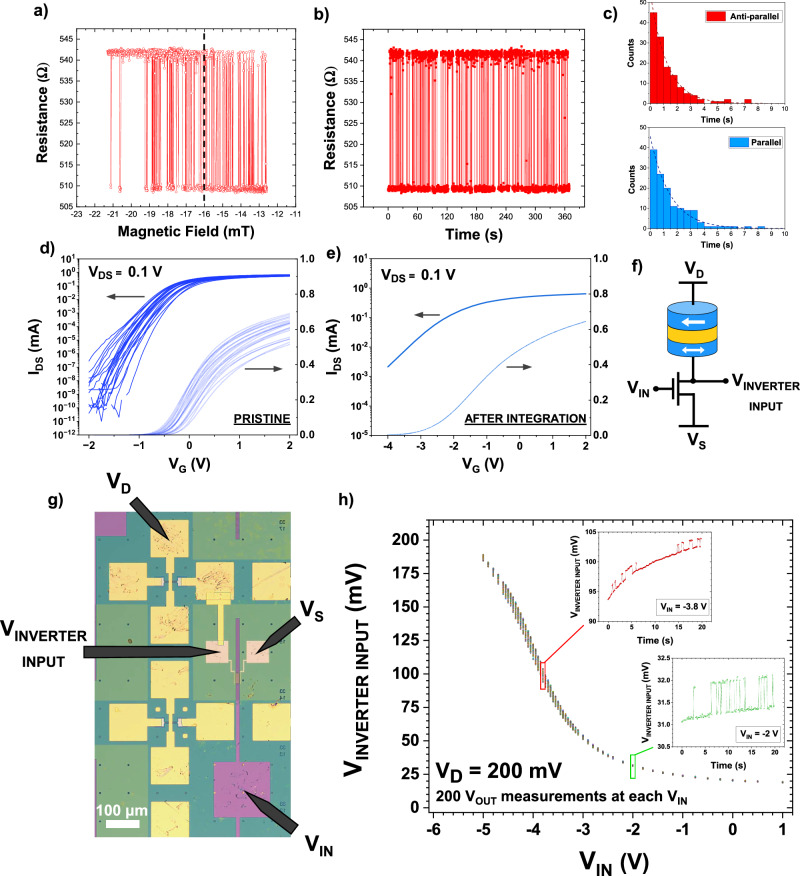
Characterization & measurement of the interconnected on-chip p-bit core. **a** Minor loop of the stochastic MTJ used in the p-bit, with the 50–50 point indicated by the dashed line. **b** Graph showing the resistance fluctuations of the stochastic MTJ when biased at the 50–50 point. **c** Histogram data for the antiparallel- and parallel-state dwell times, used to extract the mean dwell time in each state. **d** Graph showing the device characteristics of the pristine MoS_2_ FETs using the interdigitated contacts design, before being connected to the stochastic MTJs. **e** Graph showing the device performance of the FET used in the on-chip p-bit core. The degradation was observed after integrating the FET with the stochastic MTJ device. **f** Schematic of the design for the on-chip p-bit core demonstration. **g** Optical microscope image showing the interconnected on-chip device and the probes used for measurement. **h** Graph showing the operation of an on-chip p-bit core, with an output (*V*_INVERTER INPUT_) that exhibits stochastic fluctuations that are tunable with the modulation of the FET gate voltage (*V*_IN_). The observed fluctuations are obtained via repeated measurements of V_INVERTER INPUT_ at each *V*_IN_. These fluctuations are shown in the inset figures, with time-series data of the fluctuations for *V*_IN_ = −3.8 V (red inset) and for *V*_IN_ = −2 V (green inset).

Figure 3a shows the minor loop of the stochastic MTJ used in the integrated p-bit. The dashed line at −16 mT indicates the 50–50 point at which the device spends an equal amount of time in the AP- and P-state. All further measurements for this device are performed at this 50–50 point to ensure the MTJ’s resistance output (Fig. 3b) is truly random. As this is an intrinsically Poisson process, fitting the histograms of the AP- and P-state dwell times (Fig. 3c) with an exponential envelope yields the average dwell time in each state (${\tau }_{{AP}}$ and ${\tau }_{P}$, respectively)^20,39^. The dwell time of this device, a quantity that determines the speed at which a p-bit may operate, is calculated as the harmonic mean of ${\tau }_{{AP}}$ and ${\tau }_{P}$ and is 695 ms (details on the dwell time extraction and the quality of randomness can be found in Supplementary Note 2).

The transfer characteristics of 24 as-fabricated IDT ML MoS_2_ FETs are seen in Fig. 3d, showing a narrow variation in the threshold voltage, while the benefits of the IDT structure are seen in the high-current levels and on/off ratios. The on-current level is around 0.6 mA at *V*_DS_ = 0.1 V and the on/off ratio is around ~10^10^, with a minimum subthreshold slope (SS) around 94 mV/dec. Note that the scaled devices operate at gate voltages on the order of ~1 V, which is critical for the ultimate p-bit implementation to ensure that *V*_IN_ and *V*_OUT_ are identical.

Following the characterization of devices, a Ti/Au interconnect is fabricated between the MTJ- and MoS_2_-FET pair observed to have the best resistance match and stochastic signal. It is observed, however, that after the integration of MTJ and FETs, there is a degradation in the transistor performance, as shown in Fig. 3e, including degraded on-off ratio and SS. This is not a result of connecting the FET with the MTJ, but likely due to process-induced trap charges in the HfO_2_ gate oxide that produced an aging effect, whereby the FET characteristics were observed to degrade over time for this device^40^.

A circuit schematic of this 1T-1MTJ p-bit core is shown in Fig. 3f, while an optical microscope image of the finished device is shown in Fig. 3g. Figure 3h shows the output, *V*_INVERTER INPUT_, as a function of the input (FET gate) voltage, *V*_IN_. *V*_D_ = 200 mV was used to avoid excessive stress to the MgO barrier and to prevent damage to the MTJ observed at larger current densities. (To better understand the choice of *V*_D_ and the impact of large current densities through the MTJ, see Supplementary Note 3).

For this measurement, the MTJ is biased at its 50–50 point (as seen in Fig. 3a), and *V*_INVERTER INPUT_ is measured 200 times at each input voltage value, *V*_IN_, to demonstrate the impact of the stochastic fluctuations on the p-bit core’s output.

To compensate for the transistor degradation in the interconnected p-bit core, *V*_IN_ had to be significantly increased, which will not be required in a further optimized p-bit implementation. At large negative *V*_IN_, when the transistor is in its highly resistive OFF-state, the potential at *V*_INVERTER INPUT_ is close to *V*_D_. Increasing *V*_IN_ yields a decrease in the transistor’s resistance, resulting in a reduction in *V*_INVERTER INPUT_ as the transistor approaches its threshold voltage, *V*_TH_.

For this device, the leftward shift of the degraded transistor’s threshold voltage, *V*_TH_, results in a leftward shift of the overall sigmoid while the degradation in the transistor’s off-state resistance (shown in Fig. 3e) results in the output not being fully pinned to *V*_D_ (see Supplementary Note 5 for off-chip p-bit core implementations with better resistance-matching and better *V*_IN_-*V*_OUT_ matching between the constituent MTJ-FET pair, illustrating that the non-idealities in the on-chip demonstration discussed here are a result of process modules and not a fundamental issue).

The impact of the MTJ’s fluctuations also becomes increasingly clear in the p-bit core output as *V*_IN_ is increased, with the magnitude of fluctuations observed at a maximum when the resistances of the transistor and the MTJ are approximately equal, and an equal voltage is dropped across both components. The red inset in Fig. 3h reveals a significant voltage drift in the output due to charge traps from the degradation of the transistor gate oxide and its impact on the subthreshold slope.

A further increase in *V*_IN_ to the transistor’s ON-state, where the resistance of the transistor is less than that of the MTJ, sees the output approach 0 V. The output here still shows the fluctuations from the MTJ, but at a much smaller scale (green inset, Fig. 3h). This is beneficial as any STT-pinning effects from the large currents at this input voltage_,_ that could act to potentially bias the 50–50 fluctuations of the MTJ, do not significantly impact the output of the p-bit core (Supplementary Note 3 shows how large current densities through the MTJ can result in STT-pinning).

In this way, this demonstration of a scaled on-chip p-bit core is shown to produce the desired sigmoidal output with the tunable stochasticity that is required for probabilistic computing. As an individual device demonstration, and in comparing this design to a pure CMOS implementation, a high-quality tunable random number generator would require orders of magnitude more transistors/components than that which is experimentally demonstrated on-chip here^24^.

A desirable feature of the sigmoid is that it is centered around *V*_IN_ = *V*_D_/2, such that *V*_IN_ and *V*_OUT_ may be of similar scales, and the output of one p-bit may be fed into the input of another p-bit to create correlated p-bit networks. This may be achieved by implementing a dual-gated transistor design, whereby the threshold voltage may be shifted to the desired region through the application of an additional top-gate voltage (demonstrated in Supplementary Note 4).

This demonstration also illustrates the impact the transistor has on the p-bit’s output. For example, the subthreshold slope (SS) determines the steepness of the sigmoid (a steeper SS would yield a steeper sigmoid), and how well the transistor is resistance-matched with the MTJ impacts the *V*_D_ range over which the output sigmoid spans and if the output can be pinned. Moreover, the location of the threshold voltage is critical in determining the centroid of the overall sigmoid (as shown in Supplementary Note 5).

### Influence of MTJ characteristics on the p-bit output

**Fig. 4:**
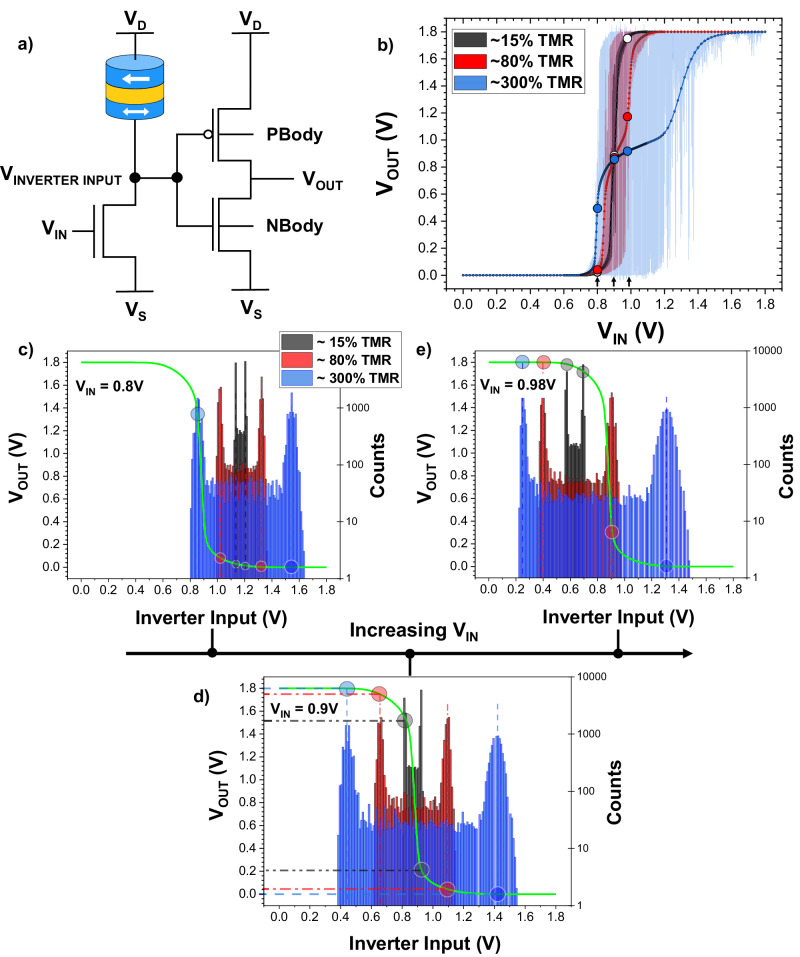
Influence of the MTJ’s TMR on the p-bit output. **a** Diagram of the p-bit circuit implemented in Cadence software for circuit simulations, with additional body bias points available for tuning of the inverter’s PMOS and NMOS transistors (labeled as PBody and NBody, respectively). **b** Graph showing the outputs of p-bits made with MTJs of differing TMRs. The dotted line shows the time-averaged output at each input voltage, while the shaded backgrounds show the instantaneous response for each p-bit. It is observed that an MTJ with too large a TMR is likely to cause undesirable plateaus in the p-bit output. **c**–**e** Graphs showing the distribution of voltages at the inverter’s input (histogram data, right axis) for the different TMR devices. The vertical and horizontal dashed lines (in Fig. 4d) demonstrate how these *V*_INVERTER INPUT_ values interact with the voltage transfer curve of the inverter (green curve, left axis), with the inverter input value (*V*_INVERTER INPUT_) being translated to an instantaneous output value that contributes towards the overall averaged *V*_OUT_ signal. This is shown for specific p-bit input voltage values of **c** *V*_IN_ = 0.8 V, **d** *V*_IN_ = 0.9 V, and **e** *V*_IN_ = 0.98 V.

To study the impact of an MTJ’s characteristics on a p-bit’s output, experimental data from stochastic MTJs are used as input for circuit simulations, conducted using the Spectre Simulation Platform. A 3T-1MTJ model of the p-bit is used (Fig. 4a), with additional bias points available at the body bias for the N-channel metal-oxide-semiconductor (NMOS) and P-channel metal-oxide-semiconductor (PMOS) transistors of the inverter for tuning of inverter characteristics (further information about data handling, and the transistors that are part of the p-bit circuitry, is provided in Supplementary Note 1).

Two key properties of an MTJ are investigated: the MTJ’s TMR and the MTJ’s distribution of resistance states. An ideal p-bit output in a 3T-1MTJ configuration would be a smooth sigmoidal function with a wide region of fluctuations, at the center of which are rail-to-rail fluctuations that could be used to drive other p-bits in a network of such devices.

Figure 4b shows the p-bit output for three MTJs fluctuating at the same frequency but with TMR ratios scaled to different values (Supplementary Note 6 describes how this TMR-scaling was performed using actual measured MTJ fluctuations). The dotted line shows the time-averaged *V*_OUT_ at each *V*_IN_, while the shaded background shows the instantaneous output as *V*_IN_ is swept linearly from 0 to 1.8 V.

The largest TMR device (300%, blue) has the widest stochastic region and rail to rail fluctuations but also shows a plateau in the time-averaged curve. These plateaus, or the pinning of the output over a range of input voltages, are non-ideal for concatenation purposes as they reduce the tunability of an individual p-bit’s fluctuations with changes in its input from other p-bits in the network.

To quantify the degree of plateau, only the central region of stochastic fluctuations is used; this is defined as the *V*_IN_ voltage range which corresponds to the middle 90%-interval of the averaged p-bit output, or the region between *V*_OUT_ = 0.18 V and *V*_OUT_ = 1.62 V. For the 80% TMR device, this corresponds to *V*_IN_ = 0.83 V and *V*_IN_ = 1.00 V, respectively. These points are used to define the “ideal” gradient, describing a line that spans these points and corresponds to an averaged p-bit output that would be consistently tunable and devoid of plateaus in the stochastic region. A plateau is defined as any point within the averaged p-bit output where the instantaneous gradient is less than 50% of the “ideal” gradient (See Supplementary Note 9 for more information on quantifying the plateau in the p-bit output).

Using these definitions, it is observed that the 300% TMR device has 67% of the stochastic region formally defined as a plateau, where little tunability is observed in the averaged output.

In contrast, the smallest TMR device (15% TMR, black) has no major plateaus within the central stochastic region but has a narrower range over which the fluctuations are visible (with the middle 90%-interval of the stochastic output being measured as between *V*_IN_ = 0.87 V and *V*_IN_ = 0.93 V).

This is undesirable as it limits the *V*_IN_ range in which usable fluctuations are observed, with the p-bit output primarily in the output-low or output-high state. To understand this behavior, consider Fig. 4c–e.

Figure 4c shows, for increasing *V*_IN_ applied to the transistor’s gate, the distributions of values at the inverter’s input for each of the p-bits made with MTJs of differing TMRs, along with the voltage transfer curve (VTC) of the inverter (overlaid in green). The largest TMR device (300%, blue), with the largest resistance fluctuation, has the widest spread of values for Inverter Input, while the smallest TMR device (15%, black) has the narrowest distribution (Supplementary Note 7 provides further explanation of these voltage distributions).

For *V*_IN_ = 0.8 V (Fig. 4c), the value at the inverter’s input is centered around *V*_INVERTER INPUT_ ≈ 1.2 V, such that the *V*_OUT_ is within output-low, i.e., close to zero, on the VTC for both the 15% and 80% TMR. However, the 300% TMR device has a sufficient number of states in the bottom arm of its *V*_INVERTER INPUT_ distribution (blue) that is in-between the noise margin regions of the inverter’s VTC, such that the average *V*_OUT_ for the 300% TMR device is shifted to a larger value of ~490 mV (Fig. 4b).

As *V*_IN_ is increased, the transistor connected to the stochastic MTJ becomes more conducting, and the center of the distributions shifts to smaller *V*_INVERTER INPUT_ values. For *V*_IN_ = 0.98 V (Fig. 4e), the 15% TMR device (black) has inverter input values such that it interacts primarily with the output-high section of the VTC, giving an average *V*_OUT_ that is pinned close to 1.7 V (Fig. 4b).

In contrast, the 300% TMR device has a larger range of inverter input values that spans between the noise margin regions of the inverter. This results in the plateau effect, where changing the input voltage does not yield a meaningful change in average *V*_OUT_ as the TMR is large enough for the distribution of inverter input values to span both the output-high and output-low regions of the VTC for a range of *V*_IN_ values.

To summarize, the smaller the TMR, the smaller the section of the VTC that is sampled by the inverter input distribution, and the smaller the range of *V*_OUT_ over which the values are averaged. This results in a smoother averaged output that is more sigmoidal and less prone to plateauing. However, the *V*_IN_ range over which the stochastic fluctuations are observed is small, limited to the range between the output-high and output-low regions of the VTC, where the gain is non-zero. This means that for a small TMR device, rail-to-rail fluctuations are not observed at all. Although it has been shown that rail-to-rail fluctuations are not necessary for the entire fluctuating range^26^, the diminished output fluctuation range would make it difficult to form networks with small-TMR p-bits due to the insufficient voltage drive it would provide to the next p-bit. A large TMR device is good for attaining rail-to-rail output voltages, such as at *V*_IN_ = 0.9 V (Fig. 4d) where the 300% TMR device shows an output spanning 0 to 1.8 V, but is prone to the plateauing effect if the device’s inverter input distribution spans the output-high and output-low regions of the VTC for an extended range of *V*_IN_ values.

This is a key finding: for a given inverter, the TMR should not be too high such that it spans the output-low and output-high regions of the inverter for a large *V*_IN_ range. Similarly, a “perfect” inverter that has an infinite gain would be undesirable for p-bit applications, as even an MTJ with a small TMR would have a step-like plateau in the output.

These plateaus are particularly problematic when interconnecting p-bits to form p-circuits. In Kaiser et al.^21^, a circuit of 5 p-bits, made with non-ideal perpendicular MTJs (in an example of off-chip integration), is used to emulate a Full Adder circuit. The performance of this non-ideal p-circuit (in which the constituent p-bits had, on average, 51% of their central stochastic range within a plateau region) is compared to an ideal p-circuit (made of p-bits devoid of plateaus in their output) (see Supplementary Note 9 for further information). It was found that the non-ideal p-circuit took twice as long as the ideal p-circuit to reach the ground state solution, demonstrating that the plateaus in an individual p-bit’s output can have a direct impact on the performance of the wider p-bit network.

Another characteristic that affects the p-bit’s output is the MTJ’s distribution of states. An MTJ with a very bimodal distribution is more prone to plateaus in the output^41^, especially if the TMR is large enough for the fluctuations in the inverter’s input to sample both output-high and output-low regions of the VTC. In contrast, an MTJ with a very continuous distribution, with the ideal being a uniform distribution between *R*_P_ and *R*_AP_, would sample each value of the VTC equally and would give a much smoother sigmoidal output.

**Fig. 5:**
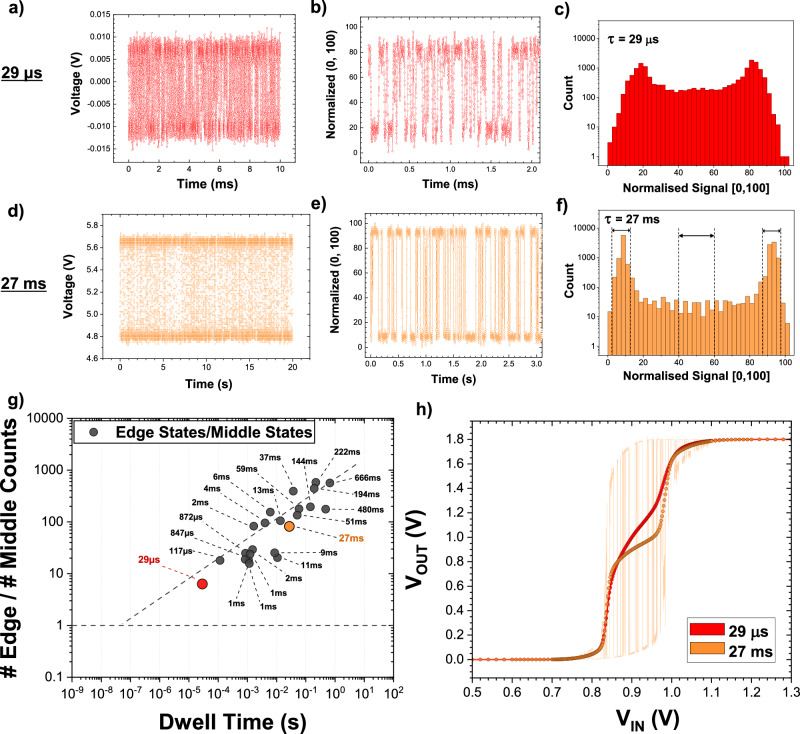
Resistance distributions of stochastic MTJ devices. **a** Data for a stochastic device with a dwell time of 29μs, showing the raw signal as measured at the oscilloscope, **b** the normalized resistance data, zoomed in to show the fluctuations are well-sampled, that are used to create **c** the device’s normalized resistance histogram. **d**–**f** Stochastic device data, as described previously, for a device of a slower dwell time of 27 ms, showing correspondingly fewer “middle states” in the normalized resistance histogram. The dashed lines and arrows represent the 8 edge-state bins and 8 middle-state bins used to calculate the “distribution factor”. **g** Graph showing the “distribution factor” of 23 stochastic MTJ devices, a quantity defined as the number of edge-state counts divided by the number of middle-state counts in an MTJ’s normalized resistance histogram, as a function of their measured dwell times. The dotted line is a guide to the eye, showing the predicted behavior for even faster devices made with this material stack. **h** Graph showing the averaged output for two p-bits that use the same inverter and transistor but with MTJs of different dwell times and distributions. Each point on these dot-line curves is produced by fixing the p-bit’s input voltage (*V*_IN_), allowing the MTJ to fluctuate over time, and then finding the average of the series of output (*V*_OUT_) values produced.

A further key finding of this work is that there appears to be a correlation between the distribution of resistance states and the speed at which these in-plane MTJs fluctuate. To quantify how bimodal a MTJ’s resistance fluctuations are, a new figure-of-merit, the “distribution factor”, is introduced. Using the normalized resistance output of an MTJ, histograms are created where the counts in the 8 edge-state bins are divided by the 8 middle-state bins. For statistical significance, the total number of data points is the same in each data set. Figure 5a–c, d–f show this process for two MTJs of different dwell times (*τ* = 29 μs and *τ* = 27 ms, respectively).

Figure 5g shows this distribution factor calculated for 23 stochastic MTJs, made with the same stack material, with dwell times spanning orders of magnitude (Supplementary Note 8 explains in greater detail why it is meaningful and justified to use the distribution factor as a key metric).

It is observed that the faster the MTJ fluctuates, the more middle states there are in the resistance distribution, and the less bimodal the distribution is. One possible explanation for this is that this distribution factor, which compares the number of counts of the edge states to middle states in the resistance distribution of an MTJ’s stochastic fluctuations, is representative of the amount of time the MTJ’s free layer spends in the P- or AP-state (the edge-state counts) compared to the amount of time the free layer spends in transitioning between them (the middle-state counts). This transition time is dependent on material properties of the MTJ stack layers, such as the effective perpendicular-anisotropy field, and for in-plane MTJs is theorized to be in the range of approximately 1–10 ns^32^. Therefore, the smaller the energy barrier, the smaller the dwell time in the P- or AP-state, while the transition time is relatively unaffected (with a change in the energy barrier size). Thus, the smaller the dwell time, the fewer the edge states relative to middle states, which correlates to a smaller distribution factor.

Considering Fig. 5g, the dotted line is a guide to the eye which suggests that for this material stack, a uniform distribution with equal edge- and middle-state counts would be achieved for MTJs with fluctuations in the tens of ns regime. This correlation suggests that a faster device, with a more continuous distribution, would yield a smoother sigmoidal output.

This is tested with the two devices of different dwell times, *τ* = 29 μs and *τ* = 27 ms, that are scaled to the same TMR, and using the same inverter (Fig. 5h). Using the same method as previously described to quantify the plateau, the slower device (27 ms, orange) has a wider plateau region with 59% of the central stochastic region (between *V*_IN_ = 0.83 V and *V*_IN_ = 1.00 V) identified as having a gradient less than 50% of the “ideal” gradient. In contrast, the faster device (29 μs, red), which has a smaller distribution factor and is less bimodal, shows only 19% of the central stochastic region as being a plateau.

Moreover, considering the severity of the plateau, the slower device’s plateau region is shallower than the plateau in the output of the faster device, resulting in the slower (more bimodal) device having an output that not only has a wider plateau region but also one that is comparatively less tunable in the central operating region.

This is another key finding in that a faster MTJ has a two-fold advantage: firstly, the faster the fluctuation and speed of random number generation, the faster the p-bit may operate asynchronously, and secondly, the faster the MTJ, the more uniform the distribution of states is observed to be, and the more ideal the p-bit’s output is. Thus, it is this interplay of the MTJ’s TMR and the distribution of states, along with the inverter’s properties, that can determine how ideal a p-bit’s output is.

### Influence of inverter characteristics on the p-bit output

**Fig. 6:**
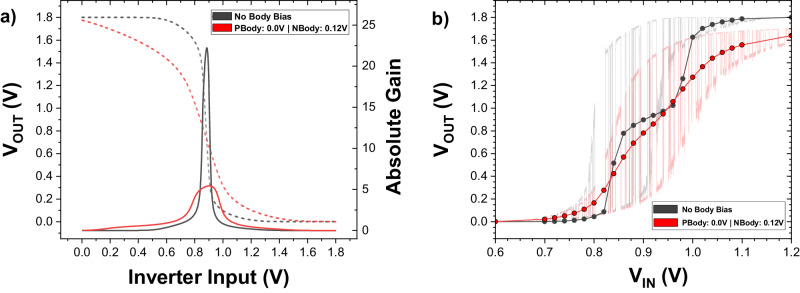
Influence of the inverter’s characteristics on the p-bit output. **a** Graph showing the voltage transfer curve (dotted line, left axis) and the absolute gain curve (solid line, right axis) for an untuned inverter (black) and an inverter that is tuned (red) to artificially lower the gain (by application of a body bias to the NMOS transistor in the inverter). **b** The averaged p-bit output, *V*_OUT_, as a function of the input voltage, *V*_IN_, for the untuned (black) and lower-gain inverter (red). Each point on the dot-line curve represents the averaged value of the p-bit’s output, obtained by fixing the input voltage (*V*_IN_) and averaging the output (*V*_OUT_) over the series of values produced due to the fluctuating MTJ resistance.

The inverter also offers a degree of control over the p-bit’s output. Figure 6a shows the voltage transfer curve (VTC) for two inverters: one without applied body bias, called “pristine” (black curve), and the other which has been tuned, through the application of a positive body bias to the NMOS FET, to have a smaller gain (red curve).

Using the same MTJ (with a dwell time of $\tau=27{{{{{\rm{ms}}}}}}$) and transistor, Fig. 6b shows the impact of this inverter tuning on a p-bit’s output: the tuned inverter (red), with the smaller gain, shows a smoother sigmoid while the pristine inverter, with the larger gain, shows a more pronounced undesirable plateau in the output. This is because for a given MTJ with a bimodal distribution, the distribution of voltages at the inverter’s input is less likely to span the output-low and output-high regions for an extended range of *V*_IN_ (the cause of the undesirable plateaus) if the VTC is shallower and the gain is small.

However, the tuned inverter also suffers from a degradation in the noise margin, seen in Fig. 6a, which decreases the size of the p-bit’s output fluctuation range. This is because the body bias at the NMOS transistor shifts its threshold voltage, lowering the channel resistance and making it harder to pin to output-high, *V*_D_, for large *V*_IN_.

This issue could be mitigated by using a more aggressively scaled technology node for the inverter than the 180 nm-node used here. A 14nm-ultrascaled Fin-FET inverter (as used in previous p-bit simulation work^26,41,42^), which provides a more piecewise-linear VTC that offers a lower gain (for a smoother sigmoidal output), and a wide-noise margin to pin the output to *V*_D_ at high-input voltages, would be desirable.

## Discussion

In this work, the experimental realization of an on-chip p-bit core is demonstrated, using a stochastic in-plane MTJ interconnected with a 2D-MoS_2_ transistor in a 1T-1MTJ structure. Through experimental demonstration and circuit simulations, it is shown how each component of the p-bit influences the overall output.

For the transistor, a good resistance match with the MTJ and a threshold voltage close to *V*_D_/2 is required to achieve a well-centered sigmoid that spans the full range of *V*_D_ and is suitable for inverter amplification.

For the stochastic MTJ, too large a TMR can cause plateaus in the inverter’s average output, while too small a TMR gives an insufficient *V*_IN_ range over which the usable fluctuations in *V*_OUT_ are observed. Additionally, it is found that the speed at which the MTJ fluctuates is crucial to the p-bit’s output: a faster MTJ is observed to have a more uniform distribution (with more middle states between *R*_P_ and *R*_AP_ edge states), and for a given inverter, this results in a smoother *V*_OUT_ sigmoid with less plateauing. A faster MTJ is also beneficial when concatenating p-bits to form a p-bit network, whereby the speed of the MTJs used can determine the speed of asynchronous operation.

For the inverter, the large gain and the steep VTC associated with the conventional 180nm-node technology used in the simulations were found to be more likely to yield undesirable plateaus in the p-bit output. A smaller gain inverter, with a piecewise-linear VTC that maintains a wide-noise margin in the input-low and input-high regions, achievable with a more scaled process, is desirable for p-bit applications.

These observations highlight how each component is crucial in determining the quality of the p-bit’s output and seek to provide design insights that can contribute towards the future goal of fully scaled on-chip p-bit networks.

## Methods

### MTJ fabrication

MTJ films are deposited using DC/RF sputtering on thermally oxidized Si substrates and, from the bottom, are Ta(8 nm)/CoFeB(2 nm)/MgO(1 nm)/CoFeB(4 nm)/Ta(4 nm)/Ru(5 nm).

These stacks are patterned into elliptical nanopillars using e-beam lithography and Ar-ion beam etching. Amorphous SiO2 is then deposited, to electrically insulate the bottom contact channel, with the etch hard mask in place as part of a self-aligned process. The hard masks are then removed using an NMP-based solvent, after which the MTJs are annealed at 300˚C for 10 minutes to improve the TMR of the finished devices^43^. After the annealing procedure, the top contacts are defined using e-beam lithography, with e-beam evaporation used to deposit Ti/Au (20/140 nm) electrodes to enable electrical measurements across the MTJ.

### 2D FET fabrication

The bottom gate electrode structure is made of a Cr (2 nm)/Au(13 nm) metal stack followed by 5.5 nm HfO_2_ gate oxide. The HfO_2_ is deposited by an atomic layer deposition (ALD) system at 90 °C. Then the ML MoS_2_ flakes are wet transferred from the original Si/SiO_2_ growth substrate onto the bottom gate electrodes and then vacuum annealed at a pressure of ~5 × 10^−8^ torr at 200 °C for 2 h. After vacuum annealing, the flakes are etched into a stripe before the interdigitated source/drain contacts are defined by electron beam lithography (EBL), and Ni (70 nm) is deposited as the contact metal by electron beam evaporation.

### Supplementary information

Supplementary Information

Peer Review File

## Data Availability

Relevant data supporting the key findings of this study are available within the article and the Supplementary Information file. All raw data generated during the current study are available from the corresponding authors upon request.
