# Supplementary material for: Experimental demonstration of an on-chip p-bit core based on stochastic magnetic tunnel junctions and 2D MoS2 transistors
Source: Nat Commun. 2024 May 15;15:4098. doi: 10.1038/s41467-024-48152-0 (PMC11096331; doi:10.1038/s41467-024-48152-0)
Supplement: Supplementary file 1 — Supplementary Information [file 41467_2024_48152_MOESM1_ESM.pdf]

Experimental demonstration of an on-chip p-bit core based on stochastic magnetic tunnel junctions and 2D MoS2 transistors – Supplementary Information

Author List: John Daniel[1,2], Zheng Sun[1,3], Xuejian Zhang[1,3], Yuanqiu Tan[1,3], Neil Dilley[1], Zhihong Chen[1,3], and Joerg Appenzeller[1,3]

Affiliations:

[1] Birck Nanotechnology Center, Purdue University, West Lafayette, IN, 47907, USA
[2] Department of Physics and Astronomy, Purdue University, West Lafayette, IN, 47907, USA
[3] School of Electrical and Computer Engineering, Purdue University, West Lafayette, IN, 47907, USA

Supplementary Note 1 – Simulation details

The simulation work presented in this paper is carried out on the Virtuoso Cadence EDA tool, using the Spectre Circuit Simulator. Both design and simulation of the p-bit are implemented using an open-source, and open-access, 180nm design kit from North Carolina State University (NCSU).

The design of p-bit consists of an MTJ connected to the drain of an NMOS transistor, forming the core tunable stochastic unit, and an inverter, which provides thresholding and amplification of the signal from the stochastic unit.

For simulation purposes, the MTJ is implemented by using a variable resistor, through which experimental data from real stochastic MTJs is fed. To prepare the data for input, the raw measurement from the oscilloscope is taken and normalized between the range [0,100], before being scaled to a certain resistance range. For the simulation work involving the comparison of different TMR coefficients, data from the same device is used and scaled to different TMR ranges. For the comparison between devices of different distributions and dwell times, data from different MTJs were scaled to the same ~80% TMR. Each resistance data set consists of 16,000 data points, with the simulator's timestep chosen such that it matches the timestep of the sampled MTJ data. This ensures the preservation of the distribution of the real MTJ's resistances. This is because data is fed into the simulator using the piece-wise-linear (PWL) algorithm that would linearly interpolate between two adjacent datapoints if the simulator's timestep is smaller than that of the sampled MTJ's resistance data. As shown in Supplementary Figure 1(a), such interpolation can alter the statistical distribution of the MTJ data and potentially impact the validity of comparison between the different MTJ p-bits.

In series with the MTJ is a NMOS transistor that is 11.25μm wide and 180nm long, designed to be resistance-matched with the MTJ in the simulation such that the full span of V_D at the inverter's input is attained.

The inverter is comprised of a PMOS transistor that is 810nm wide and an NMOS transistor that is 270nm wide, with both being 180nm long. For simulations that do not bias the inverter, the body bias of the inverter's PMOS transistor is set to 1.8V (V_{DD}) and the inverter's NMOS transistor to 0.0V (V_{SS}).

The example outputs in Supplementary Figure 1(b) and 1(c) (reproduced from Figure 1 of the main text) are obtained by sweeping V_{IN} and sampling the voltage levels before the inverter's operation, defined as "$V_{INVERTER\ INPUT}$", and after the inverter's operation, defined as V_{OUT}.

The graphs show the transient instantaneous value (red), obtained by sweeping the input voltage, V_{IN}, concurrently with the resistance data being fed through, while the dotted lines (yellow) show the average V_{OUT} value, calculated by fixing the p-bit's V_{IN} value and allowing the mimic MTJ to fluctuate over time, and averaging the output voltage over the entirety of the values produced from the resistance data available (over the 16,000 data points).

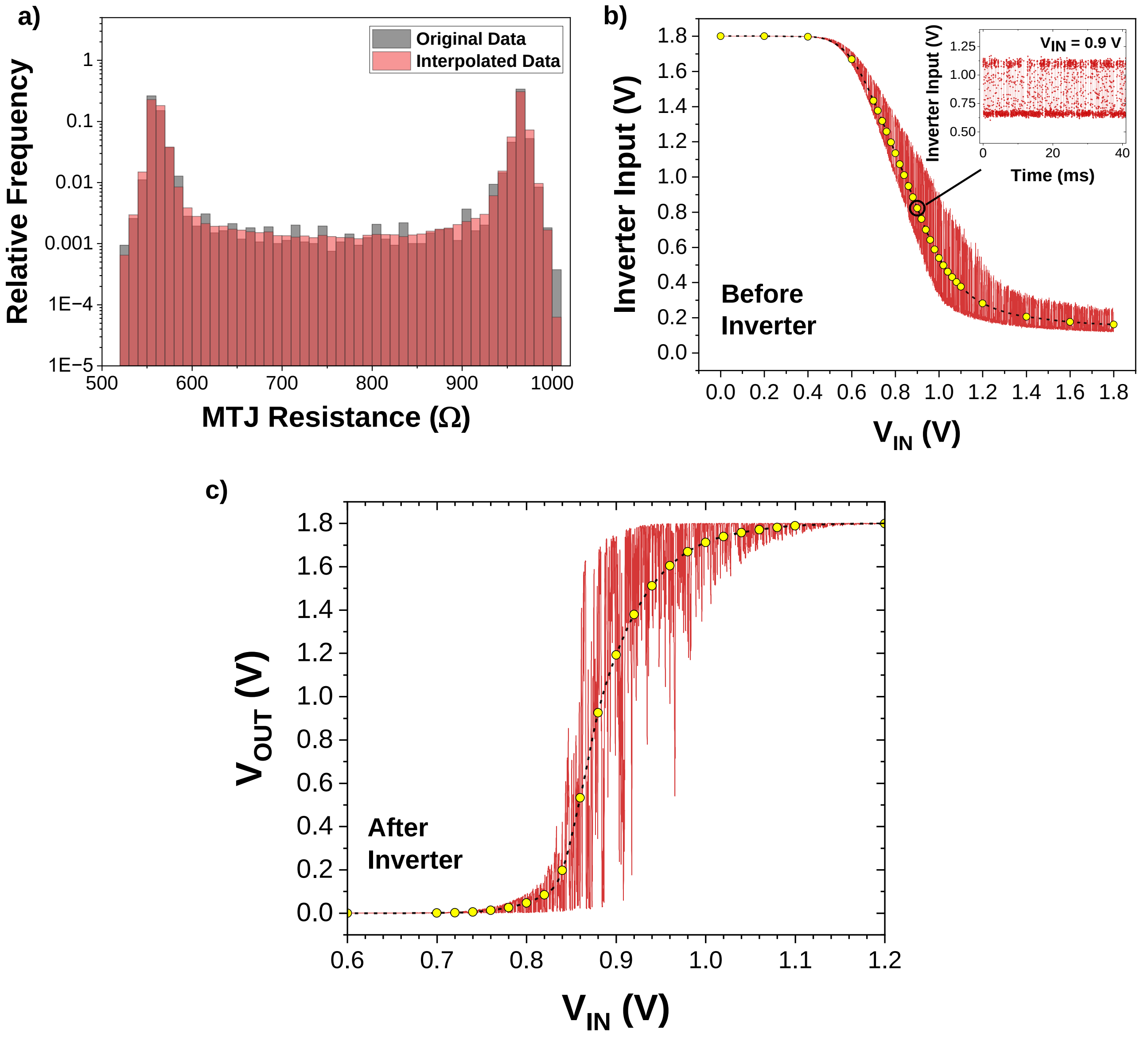

Supplementary Figure 1: Simulation Details. a) Plot showing the difference in the MTJ distribution when fed using the correct timestep ("Original Data"), and when using a too small timestep ("Interpolated Data"). b) The output sigmoid of the stochastic p-bit core, $V_{\text{INVERTER INPUT}}$, as a function of the input voltage, V_{IN}. The red line shows the transient instantaneous value of the voltage, while the dotted line shows the time-averaged value. c) A plot of the full p-bit's output, after the inverter's thresholding and amplification. The value of V_{OUT} is pinned to 0V for V_{IN} below 0.6V, and to 1.8V for V_{IN} above 1.2V, and so is not shown here.

Supplementary Note 2 – Measurement details & further characterization of stochastic MTJs & NIST randomness tests

All electrical measurements are conducted in a Lakeshore probe station equipped with an in-plane magnetic field. The characterization of the transistors, and the experimental demonstration of the core of the stochastic p-bit (shown in Section 3 of the main text), are achieved using DC measurement techniques with an Agilent HP 4155B Parameter Analyzer.

The measurements of the stochastic MTJs presented in this work are performed using AC lock-in techniques, with a SR830 Lock-in Amplifier (LIA) and a Keithley 2400, in a four-point configuration to directly measure the properties of the MTJ pillar.

Supplementary Figure 2(a) shows the time-series resistance fluctuations for the MTJ device used in the 1T-1MTJ p-bit core as a function of increasing magnetic field. At an external field of -18mT, the device "spends" most of its time in the high-resistance AP-state while at -12mT, the device is predominantly in the low-resistance P-state. The dwell-times in the AP- and P-state are plotted as a function of external magnetic field, shown in Supplementary Figure 2(b), and used to identify the field at which the MTJ spends an equal amount of time in the P- and AP-state (-16mT), defined as the 50-50 point. The slight difference in tunability of the P- and AP-dwell-times by the external magnetic field, attributed to an experimental artifact of this measurement, does not appear in similar measurements of other stochastic devices, and is not analyzed further.

Aside from the tunability, a second requirement of the p-bit core for which the MTJ is used is that the stochasticity must be random. To quantify the quality of randomness, the output of these fluctuating MTJs (at the 50-50 point) are binarized (Supplementary Figure 2(c)) and put through the NIST suite of randomness tests[1,2].

Supplementary Figure 2(d) shows the results of these tests, where a p-value greater than 0.01 denotes a success, for the binary string attained when the output is sampled approximately every dwell time (~0.6s). The results show that when biased at this 50-50 point, the MTJ is indeed a source of true randomness and is suitable as the stochastic source in the p-bit core.

This is a key demonstration that highlights how low-barrier nanomagnets, coupled with MTJs for read-out, can form low-cost and compact random number generators that fluctuate naturally from ambient thermal energy, providing a platform that is especially suitable for p-bit applications.

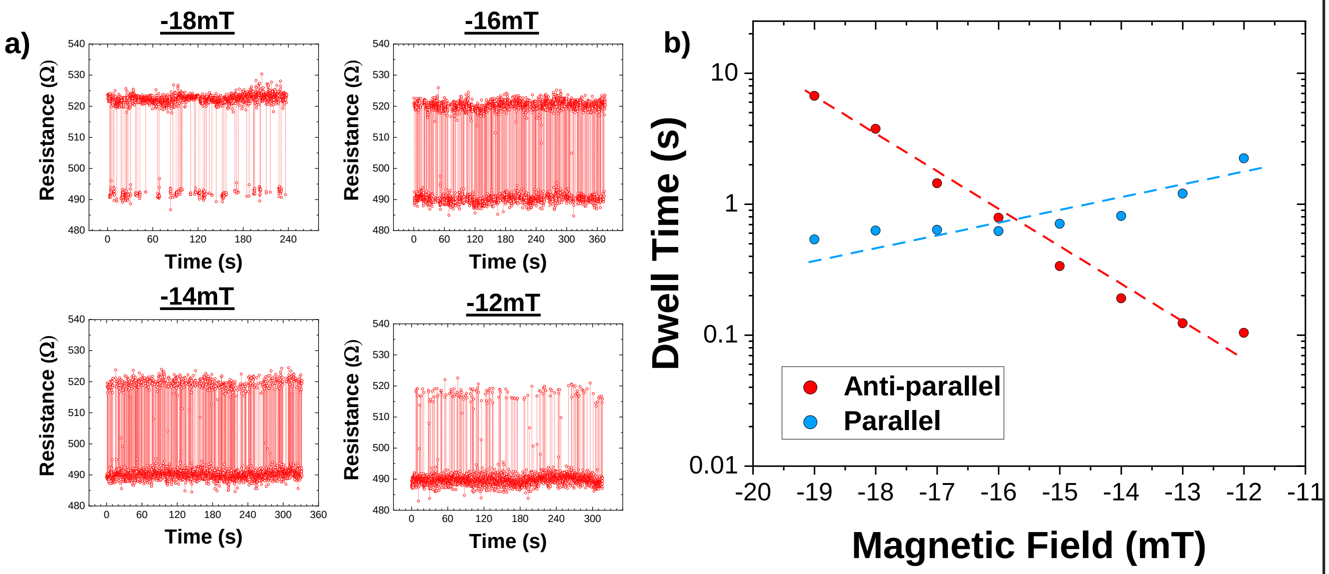

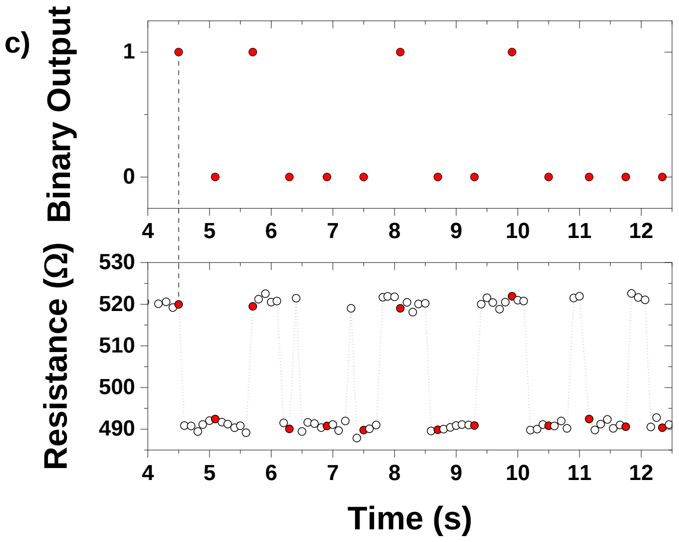

d)

Test	Passed if p-value > 0.01
Frequency	0.020 ✓
Block Frequency	0.066 ✓
Runs	0.013 ✓
Longest Runs	0.021 ✓
Discrete Fourier Transform	0.303 ✓
Non-overlapping Template	0.112 ✓
Serial Test	0.683, 0.249 ✓
Approximate Entropy	1.0 ✓
Cumulative Sums	0.039 ✓

Supplementary Figure 2: Electrical characterization of stochastic MTJs. a) Time series data of stochastic resistance fluctuations at varying external in-plane field to show tunable stochasticity. b) AP- and P- dwell times plotted as a function of magnetic field to find the 50-50 point of this MTJ. c) Process showing the binarization of the resistance fluctuations to generate the bitstream for the NIST Randomness test. d) Results from the NIST randomness tests, showing the output of the MTJ is indeed truly random.

Supplementary Note 3 – Spin Transfer Torque (STT) & current density experiments

This section presents measurements on how large drive voltages may impact p-bit performance, and further explores the impact of large currents, through the resulting Spin Transfer Torque (STT) effect, on the in-plane MTJs used in the experimental demonstration of the stochastic p-bit core.

To this end, the resistance fluctuations of the MTJ used in the p-bit core demonstration are measured in a two-point configuration, while under an external field of -16mT (to bias the MTJ at the 50-50 point). This two-point configuration includes the additional resistance of the current path from the Ta channel between the contact pads and the MTJ pillar, and better mimics the actual resistance of the MTJ branch of the p-bit core.

Supplementary Figure 3(a) displays the measured values of the fluctuation's high-resistance AP-state and low-resistance P-state as a function of applied DC voltage, and illustrates that as increasingly large biases are applied across the MTJ, the base resistance degrades. This is likely because of degradation of the thin MgO layer as the stress current is increased for increasing bias voltages[3]. Reverting to a lower voltage once the MgO layer is damaged also does not fully recover performance, but results in a smaller base resistance. This is a key reason for why a V_D of 200mV was chosen for the experimental demonstration in Section 3: the low RA-product of the thin MgO layer ($\sim 7\ \Omega\mu\mathrm{m}^2$) results in large current densities (>10^6 A/cm^2 for voltages above 200mV) that pose an increased risk of barrier breakdown during extended operation.

Indeed, for interconnected p-bits, parity is desired between the V_D and V_{IN} range such that V_{OUT} can be used to drive the next p-bit in the network. For this to be achieved, and to ensure current densities are not too large to pose a risk of barrier breakdown, it is found that MTJ area-scaling is insufficient to reach the larger resistances required if the MgO thickness is too thin and the RA-product of the resulting MTJ is too small. Increasing the base resistance of the MTJ to the $10^4\ \Omega$ range will be required for future generations of unstable MTJs to achieve the operation of p-bits at the voltage range desired.

Aside from the impact on the device resistance, the impact of large current densities through STT on the MTJ's resistance fluctuations are also considered. Supplementary Figure 3(b) shows the calculated current density as well as the probability of the high-resistance AP-state for each increasing DC voltage. This quantity is calculated by binarizing the output of the resistance fluctuations, with '0' representing the low-resistance P-state and '1' representing the high-resistance AP-state, and averaging over the binary bitstream collected. Supplementary Figure 3(c) shows the time series data of the resistance fluctuations, as well as the binary output used for this operation, at applied voltages of 200mV, 400 mV and 1V.

It should be noted that the relatively slow dwell time (~ 0.7s) of the device and the small sample time of measurement yields a probability-curve in Supplementary Figure 3(b) that deviates from the expected smooth sigmoid when studying the impact of STT on MTJ resistance fluctuations. Regardless, one can see that it requires a bias of 400 mV before a significant pinning effect on the fluctuations of this device is observed. This corresponds to a current density of $\sim 2 \times 10^6$ A/cm^2 and is consistent with the values found by others when working with the impact of STT-currents on in-plane MTJs of similar resistance[4]. This result also highlights that the 200mV chosen for the V_D in the experimental demonstration in Section 3 yields a current density that *does not*

cause pinning issues and does not unduly influence the MTJ's random fluctuations, preserving its integrity as a true random number generator.

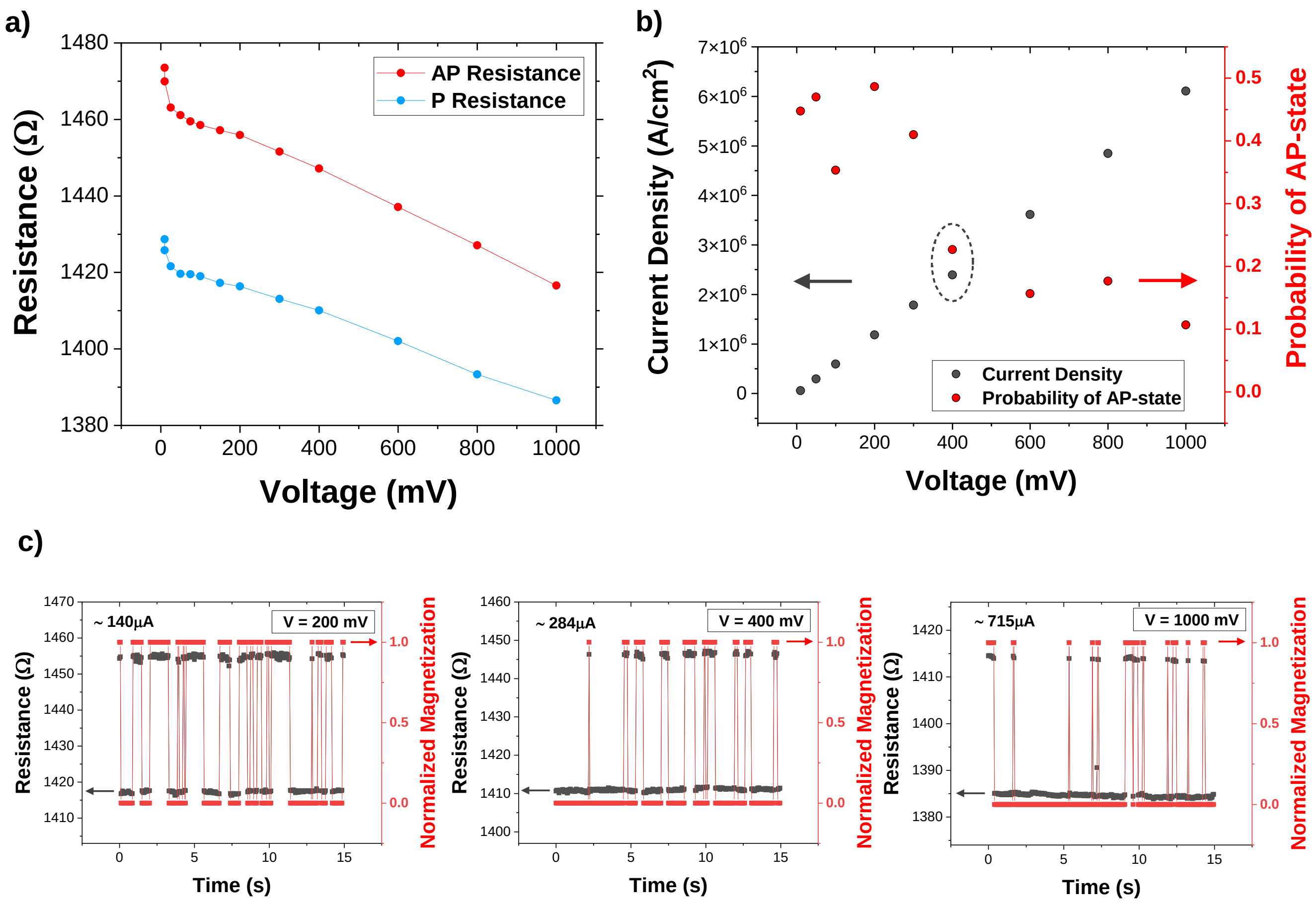

Supplementary Figure 3: Current Density & Spin Transfer Torque (STT) experiments. a) Plot showing the AP- and P-resistance levels as a function of applied DC voltage across the MTJ. b) The associated current density (left axis) and the calculated probability of the AP-state (right axis) for the resistance fluctuations plotted as a function of the applied DC bias across the MTJ. c) A series of graphs that shows the time series resistance fluctuations at the 50-50 point for increasing DC bias to show the impact of STT on the MTJ's fluctuations. Note that it is only at 400mV that the pinning effect to the P-state starts to be observed.

Supplementary Note 4 – Dual-gated FETs & threshold voltage control

To form networks of correlated p-bits, the output of one p-bit should be in the same voltage range as the input of the next p-bit. This means that after the inverter's operation, it is necessary for the output to be able to achieve near 0 to V_D fluctuations at a specific input voltage range, and for the output sigmoid to be centered around $V_D/2$ at the input.

The 1T-1MTJ design for the p-bit core demonstrated in Section 3 utilized a back-gated MoS_2 transistor that, although displaying a narrow distribution of threshold voltage, V_{TH}, when pristine, was seen to suffer degradation through an aging effect that saw a leftward shift in the resulting V_{TH} in the final integrated demonstration.

To address this, a double-gate design has been explored to offer additional control over threshold voltage and to allow a rightward shift of the threshold voltage, which controls the centroid of the sigmoidal output.

The fabrication process involves first creating the back-gated MoS_2 devices, as described in the main body of the text, after which an Al seeding layer (~1nm) is grown and an AlO_x layer is formed. Atomic Layer Deposition (ALD) of the top HfO_2 dielectric layer (11nm) is then performed at 90˚C after which the top-gate Au contacts are deposited. Supplementary Figure 4(a) shows a cross-section schematic of this double-gated MoS_2 FET. The resulting FETs are interconnected to resistance-matched MTJs across two probe-stations (Supplementary Figure 4(b)), to form an example of an off-chip p-bit core.

The transfer characteristics of a double-gated FET, shown in Supplementary Figure 4(c), demonstrate that with an increasingly negative top-gate voltage, the Fermi level is shifted towards the valence band which induces the rightward shift in threshold voltage, towards more positive back-gate voltages.

As stochastic fluctuations were not necessary for this demonstration of a double-gated p-bit core, a stable in-plane MTJ of circular design, 200nm in diameter, was chosen. The resistance vs magnetic field behavior for this device is shown in Supplementary Figure 4(d). The lack of shape anisotropy means that the magnetic moments are not defined to be colinear with the applied external field axis, and the stray field coupling of the fixed layer with the free layer ensures that at zero field, the moments are in the antiparallel high-resistance state. As the magnetic field is increased in either direction, the moments of both are brought gradually into alignment with the externally applied field, yielding a low-resistance parallel-state at high fields.

Supplementary Figure 4(e) shows the output for this double-gated p-bit core, measured at the drain of the FET, $V_{INVERTER\ INPUT}$, at different set top-gate voltages while the back-gate voltage (V_{IN}) is swept from -1.7V to +1.5V. The V_D is limited to 400mV to minimize the risk of oxide breakdown in the MTJ. Overall, it is seen that with application of increasingly negative voltages to the top-gate, the threshold voltage, and the resulting p-bit core output sigmoid, can be shifted to the right. Indeed, for V_{TG} = -2.5V, the output sigmoid is well-centered around 0V and may be pinned with the noise margin at the output-high and output-low values.

This demonstrates that a dual-gated FET design is a viable pathway to achieving threshold voltage control, resulting in a well-centered sigmoidal output that is suitable for the next-step of inverter amplification, and further offering an additional mode of control for in-situ adjustments of devices in correlated p-bit networks.

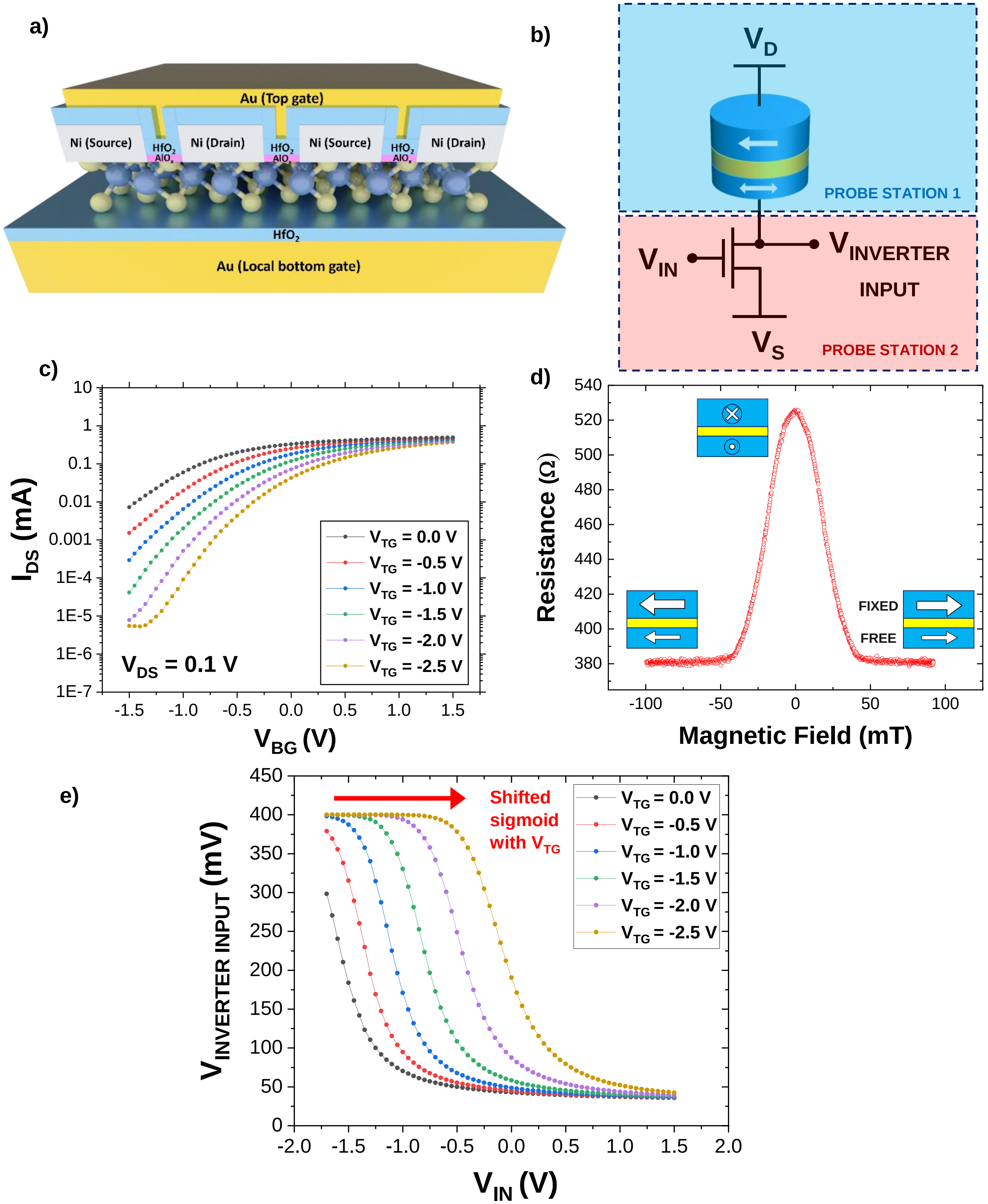

Supplementary Figure 4: Control of threshold voltage and sigmoid-center using a double-gated FET. a) Cross-section of the double-gated MoS_2 FET structure. b) Schematic of the integrated p-bit core, showing the split of devices across two probe-stations. c) FET transfer characteristics where a rightward shift in V_{TH} at increasingly negative top-gate voltages is observed. d) Four-point measurement of the MTJ pillar showing the major loop of the stable in-plane MTJ device used for this demonstration. e) Sigmoidal output for this integrated double-gated p-bit core, demonstrating that the sigmoid may be shifted with application of a top-gate voltage.

Supplementary Note 5 – Further demonstration of 1T-1MTJ p-bit core using well-resistance matched MTJs & FETs

This section covers examples of better resistance-matched MTJ & FETs and aims to further highlight how aspects of each can influence the sigmoidal output of this p-bit core structure. The p-bit core devices presented here are examples of a well-resistance matched device and a device that utilizes a fast MTJ; both devices are integrated across two probe stations in an off-chip structure, as shown in Supplementary Figure 5(a).

Supplementary Figure 5(b) shows the FET transfer characteristics, and Supplementary Figure 5(c) and 5(d) show the MTJ characteristics, for the slow but well-resistance matched p-bit core while Supplementary Figure 6(a) and Supplementary Figure 6(b) and 6(c) show the FET, and MTJ, characteristics for the second p-bit core, respectively. The minor loops in Supplementary Figure 5(c) (and Supplementary Figure 6(b)) are measured using four-point lock-in techniques across just the MTJ pillar, while the time series resistance fluctuation measurements in Supplementary Figure 5(d) (and Supplementary Figure 6(c)) are two-point measurements, measured at the MTJ's 50-50 point, that include the additional Ta channel resistance.

First, it is noted that both transistors have a V_{TH} close to zero, without top-gate control, and this results in well-centered sigmoidal outputs.

Secondly, consider the resistance-matching between the MTJ and FET for the two p-bit core devices. Supplementary Figure 5(e) shows the output of the first, well-resistance matched, device where in the off-state the FET is many orders of magnitude more resistive than the MTJ, allowing for the output to be easily pinned to V_D at low V_{IN}. In the on-state, the FET resistance reaches the $10^2\ \Omega$ range with the high current-drive provided by the interdigitated finger design, while the MTJ has a resistance in the $10^4\ \Omega$ range. The resulting voltage division means the output may be pinned effectively to 0 at large V_{IN}.

In contrast, Supplementary Figure 6(d) shows the output of a p-bit core where the resistance matching is not as ideal. In the off-state, the MTJ resistance is much less than the FET resistance, allowing the output to be pinned to V_D at low V_{IN}. However, the lower base resistance of the MTJ (in the $10^3\ \Omega$ range) means in the on-state, where the FET resistance is in the $10^2\ \Omega$ range, gives only a 10:1 resistance ratio that means that for large V_{IN}, the output does not approach 0 but rather $V_D/11 \approx 27$mV. This is still acceptable with an inverter that has a suitable noise margin but is an example of a less-ideally resistance-matched p-bit core.

Another difference between the two outputs is in the speed of fluctuation of the MTJ used in the corresponding p-bit cores. The non-ideal case here are the fluctuations from the slow MTJ, shown in Supplementary Figure 5(d), where the bimodal resistance distribution manifests as a very bimodal distribution in the $V_{INVERTER\ INPUT}$, shown in the inset of Supplementary Figure 5(e). As described in Section 4 of the main text, this can cause problematic plateaus in the overall p-bit's output.

In contrast, the faster MTJ of Supplementary Figure 6(c) has a much more continuous distribution, where the resistance values in-between the bounding low- and high-resistance states of the fluctuations are sampled more evenly, and the $V_{INVERTER\ INPUT}$ values (shown in inset of Supplementary Figure 6(d)) reflect this. This is crucial for attaining a p-bit output sigmoid that is devoid of the non-ideal plateaus described previously.

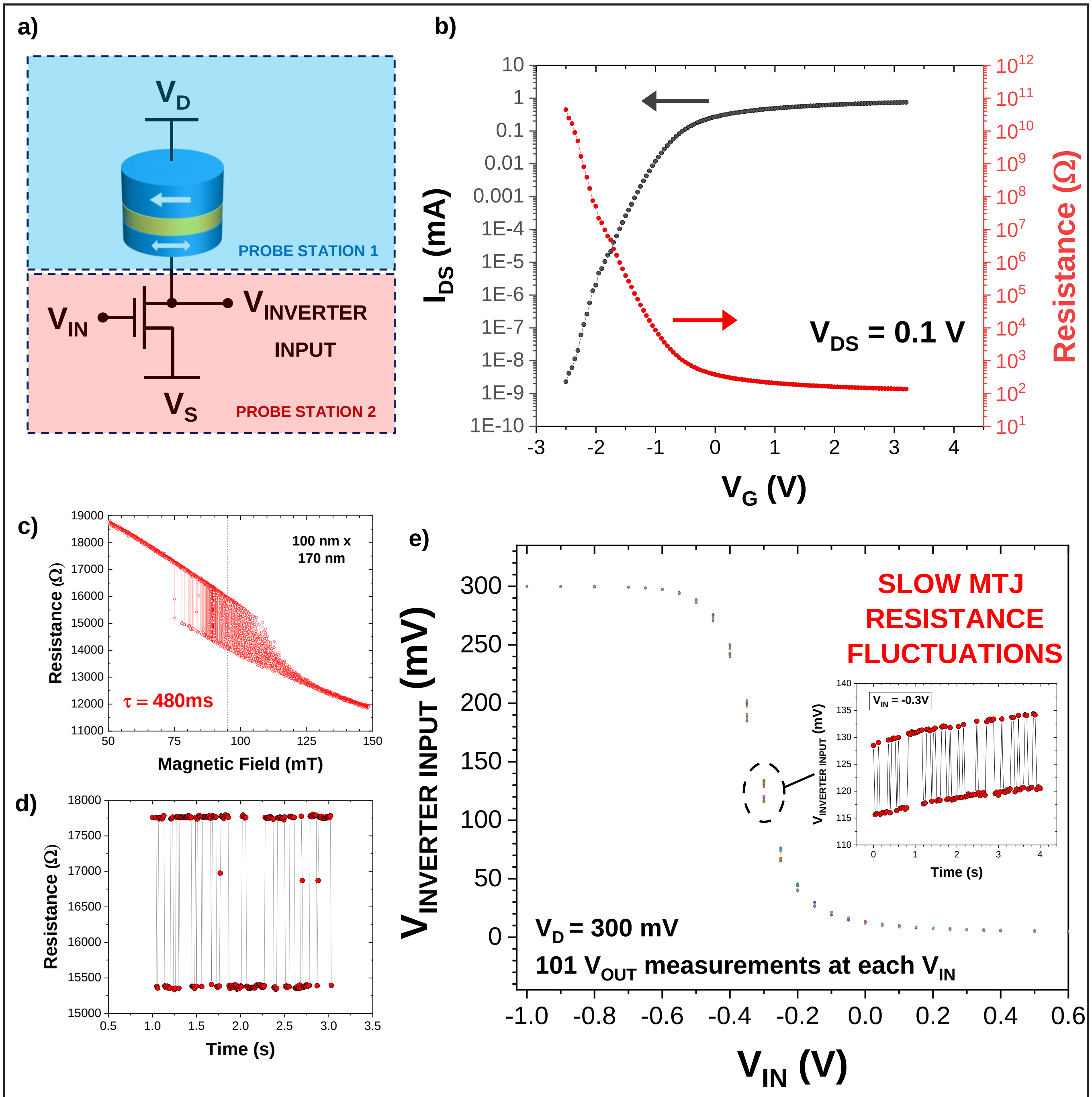

Supplementary Figure 5: Measurements of a well-resistance matched p-bit core. a) Schematic of the p-bit core, showing the split of devices across the two probe-stations. b) FET transfer characteristics, with the drain current (left-axis) and the calculated FET resistance (right-axis) plotted as a function of gate voltage, V_G, at a drain voltage of 0.1V. c) Four-point measurement of the MTJ pillar showing the minor loop and the 50-50 point. d) Two-point time-series measurements of the MTJ to demonstrate the bimodal resistance fluctuations when biased at the 50-50 point. e) Output for the p-bit core made with the bimodal MTJ, showing good pinning at V_D and 0 for low and high V_{IN}, respectively, but a bimodal voltage distribution at the $V_{INVERTER\ INPUT}$.

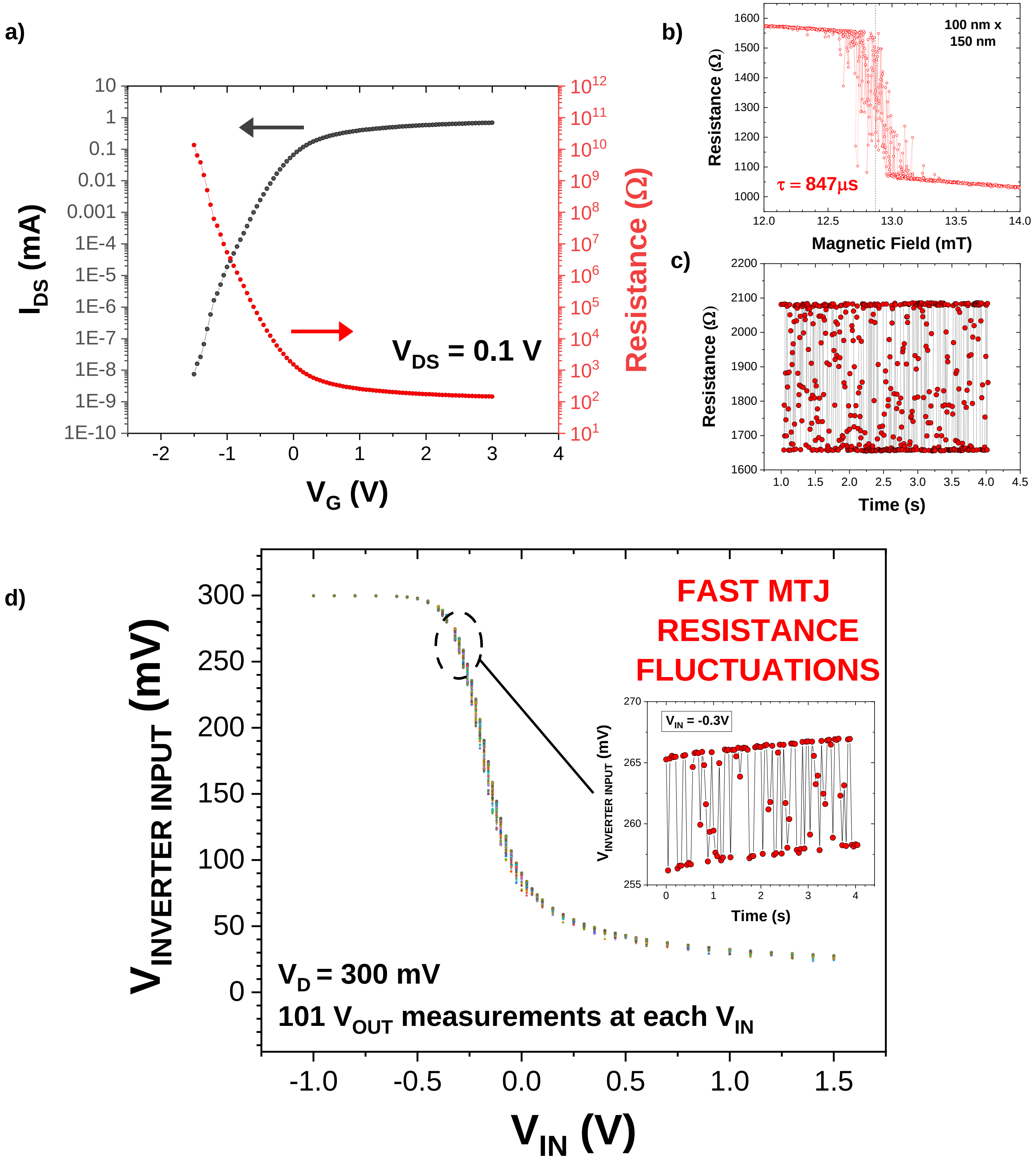

Supplementary Figure 6: Measurements of a fast MTJ p-bit core. a) FET transfer characteristics, showing the drain current (left-axis) and the calculated FET resistance (right-axis) at a drain voltage of 0.1V. b) Four-point measurement of the MTJ pillar displaying the minor loop and highlighting the 50-50 point. c) Two-point time-series measurements of the MTJ to demonstrate the continuous resistance fluctuations when biased at the 50-50 point. d) Output for the p-bit core made with the continuous MTJ, showing good pinning at V_D for low V_{IN} but the inability to pin to 0 at high V_{IN}. However, the continuous resistance distribution of the MTJ manifests as a continuous voltage distribution at the $V_{INVERTER\ INPUT}$.

Supplementary Note 6 – TMR scaling for simulation input

To understand how the Tunnel Magnetoresistance (TMR) coefficient of a MTJ affects the output of a p-bit, three different resistance ranges are chosen to yield TMR coefficients of ~15%, ~80% and ~300%. The input data is taken from time series measurements of a real stochastic MTJ device, fluctuating at its 50-50 point, and exhibiting a dwell time of 847us. The raw input is then normalized between the range of [0,100] and scaled to the three TMR ranges described previously. The resulting time-series outputs are shown in Supplementary Figure 7(a), where each output consists of 16,000 data points.

To verify that this scaling is valid, statistics are presented for 23 actual stochastic devices to map any possible correlations that could impact the validity of the TMR scaling approach employed in this work, and to see if the magnitude of the resistance fluctuations influences the dwell time.

Supplementary Figure 7(b) shows the dwell time plotted against the resistance range over which fluctuations are observed, $R_{AP} – R_P$, while Supplementary Figure 7(c) shows the dwell time plotted against the TMR of the stochastic fluctuations. As the plots show, there is no correlation between the dwell time and either of these quantities and so the TMR scaling can be performed without changing other key properties of the stochastic MTJ.

The resulting TMR distributions are shown in Supplementary Figure 7(d). It should be noted that the scaling is done to ensure the midpoint for each device is the same, i.e. 750Ω, so that the same transistor and inverter may be used in all three cases without concerns regarding resistance matching, and to preserve the overall distribution of states.

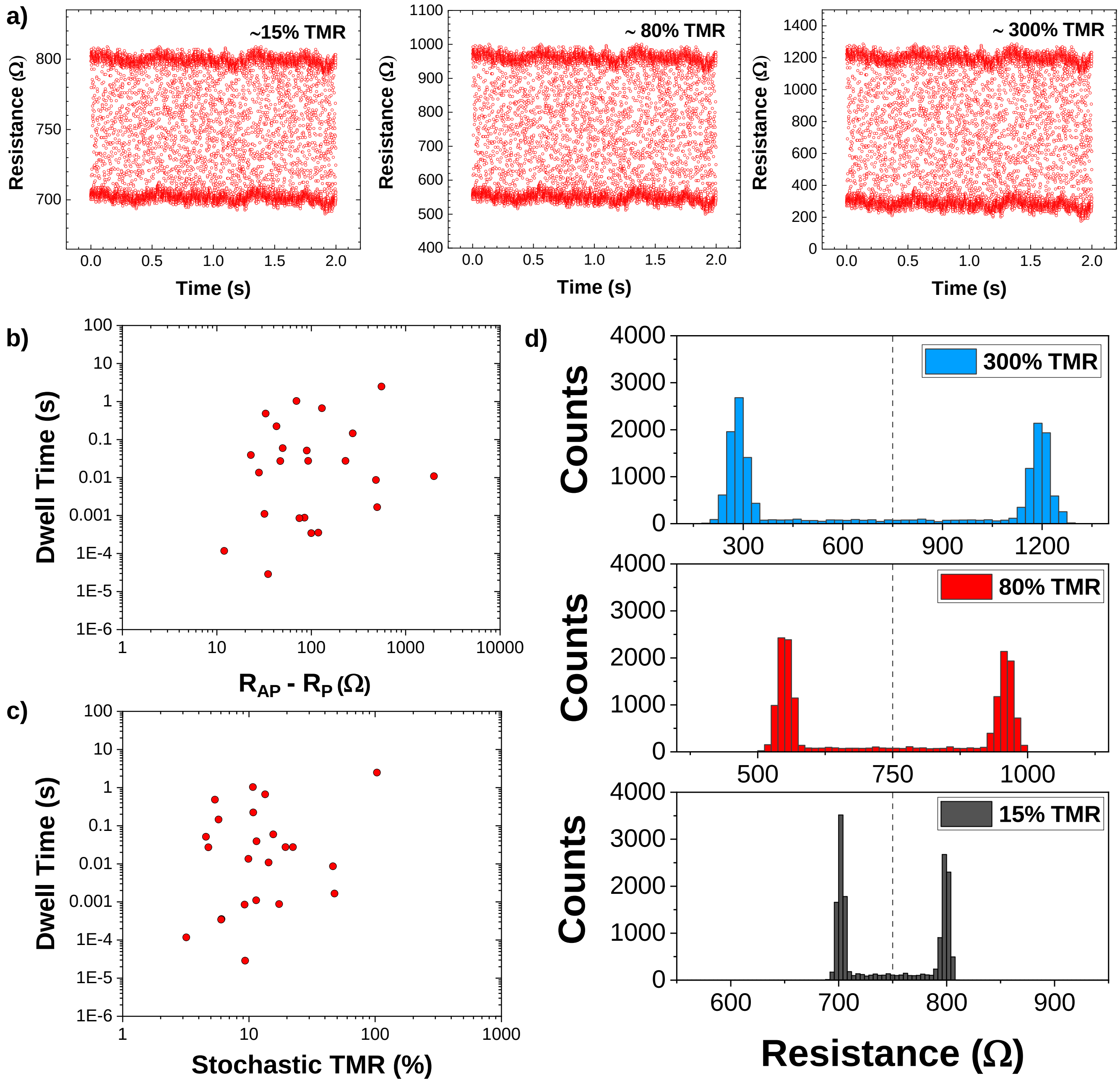

Supplementary Figure 7: Details on TMR scaling for simulation input. a) Time series data for the ~15% TMR, ~80% TMR and ~300% TMR device constructed from real experimental data of a stochastic MTJ. b) Plot displaying statistics for 23 stochastic MTJ devices, showing how the dwell time does not correlate with the size of the stochastic resistance fluctuation ($R_{AP} – R_P$). c) Plot demonstrating how the measured dwell time has no correlation with the MTJ's TMR. This suggests the scaling is a valid approach without introducing other variables that might impact the distribution/speed of fluctuation. d) Histograms for the three devices of increasing TMR used in the simulation, highlighting how the midpoint is kept at the same value so the same resistance-matched transistor and inverter may be used across all three p-bits.

Supplementary Note 7 – Explanation of voltage distribution for impact of TMR on p-bit's output

This section aims to further clarify the nature of the histograms showing the distribution of voltages at the inverter's input for the p-bits made with MTJs of different TMRs (Figure 4(c) of the main text). Three cases were considered in particular: for V_{IN} = 0.8V, V_{IN} = 0.9V and V_{IN} = 0.98V, with a focus here on explaining the broadening of the distributions, as well as the change in shape and symmetry of the distributions, as V_{IN} increases between these three cases.

A key point to note is that the MTJs in this p-bit design are primarily "noisy resistors", and only provide the stochastic behavior. As a result, their resistance values are confined to fluctuate between the R_{AP} and R_{P} values, and do not change as a function of V_{IN} (Supplementary Figure 8(a)). The tunability is obtained by modulating the gate voltage of the transistor that is in series with the MTJ, such that the voltage at the inverter's input may be tuned according to the relative resistance between, and voltage drop across, the MTJ and FET.

For a fixed input voltage (V_{IN}), the stochastic resistance fluctuations of the MTJ lead to a fluctuating drain voltage for the FET, resulting in a fluctuating current through the MTJ + FET (as shown in Supplementary Figure 8(b)).

For small V_{IN}, where the transistor is in the off-state and the resistance is much larger than the MTJ resistance, this effect is minimal, and the fluctuations are suppressed. However, as the MTJ and FET resistance are brought into similar ranges with increasing V_{IN}, the impact of the MTJ's fluctuations, and the relative change in the drain voltage of the FET, are much larger. This occurs around $V_{IN} \approx 0.8$V, as seen in Supplementary Figure 8(c) and 8(d), and helps explain the widening of the distributions as V_{IN} is further increased.

This broadening of the $V_{INVERTER\ INPUT}$ values is also seen in Supplementary Figures 8(e) and 8(f), where bigger swings in voltage drop are first observed at V_{IN} where the MTJ and FET resistances are of a similar magnitude.

These graphs, which show the output of the stochastic p-bit core before inverter amplification, also explain the apparent asymmetry in the 300%-TMR distributions at V_{IN} = 0.98V. Note that the lower arm of the distribution corresponds to the high-resistance state of the 300%-TMR MTJ. At V_{IN} = 0.98V, the ratio of MTJ resistance to FET resistance (in the low $10^2\ \Omega$ range) is such that the 300%-TMR MTJ is nearly always at a much larger resistance than the FET, resulting in more $V_{INVERTER\ INPUT}$ values that are small and an asymmetrical distribution.

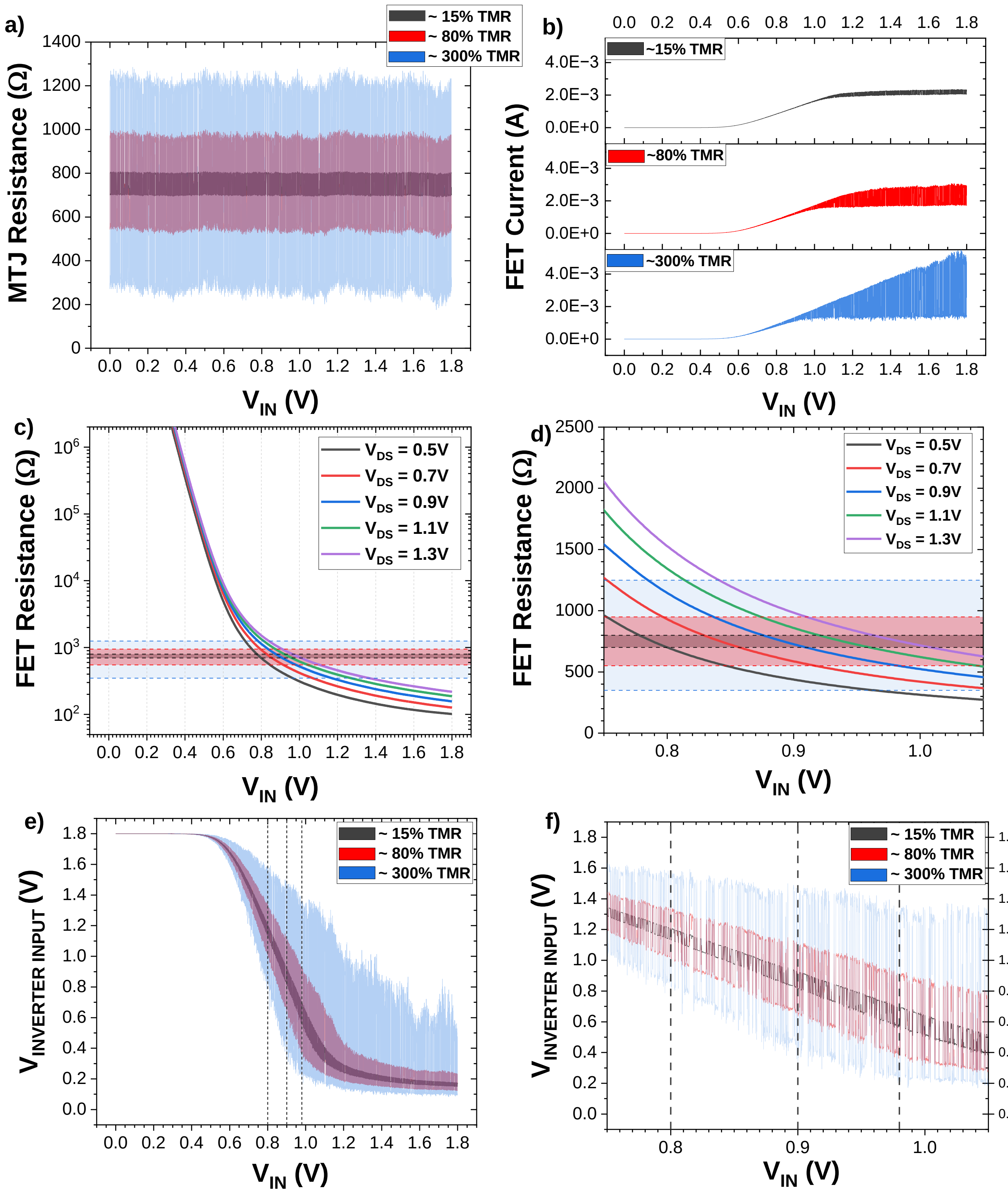

Supplementary Figure 8: Further explanation of the voltage distribution histograms for the impact of TMR on the p-bit's output. a) Plot showing how the resistance fluctuations of the three TMR-devices are invariant with V_{IN}. b) Graph plotting the NMOS FET current against the input voltage, demonstrating how the resistance fluctuations in the MTJ manifest as fluctuations in the current through the FET. c) Plot of the FET resistance, calculated at different V_{DS}, along with the MTJ resistance (colored bands) for the full V_{IN} range and d) for the narrow range shown in the histograms in Section 5 of the main text. e) Plot of the voltage at the $V_{INVERTER\ INPUT}$ point for the three TMR devices as a function of the full V_{IN} range, and f) for the narrow range of V_{IN} from which the histograms are taken.

Supplementary Note 8 – The Distribution Factor

The ‘distribution factor’ is a quantity that is introduced as a measure of how bimodal, or continuous, an MTJ’s resistance fluctuations are. It is calculated by taking the resistance fluctuations of an MTJ, normalizing the output to the range [0,100], and creating a histogram distribution of the resulting normalized output. Care is taken to ensure the same size of data set is used for each stochastic device (16,000 resistance data points for each distribution), and that equal bin sizes are used (to make the comparison of counts statistically useful).

The counts in the 8 ‘edge’-state bins (which represent the counts in the P- and AP-resistance states) are divided by the counts in the 8 ‘middle’-state bins (which represent the counts in-between the bounding P- and AP-state) to give a number defined as the ‘distribution factor’. A value that is very large indicates a very bimodal distribution, whereby the number of edge states are far more numerous than the number of middle states, whereas a small number indicates a more continuous distribution where the number of edge and middle states are similar. An ideal ‘uniform’ distribution, where the number of edge and middle states is identical, would be represented by a distribution factor of 1.

A key finding of this work is that there appears to be a correlation between this distribution factor, or the distribution of resistance states, and the dwell time of the stochastic device. A faster device is observed to have a more continuous distribution, with more middle states, and a correspondingly smaller distribution factor. This is important as a more continuous distribution is shown to be desirable, with the resulting p-bit output having fewer non-ideal plateaus the more continuous the distribution is.

One potential worry is that this quantity is a function of the sampling time of measurement. Faster devices require faster sampling times (shorter $\Delta\tau$ between measurement points) to accurately capture their fluctuations, and this could account for the correspondingly smaller distribution factor as the number of middle states would be inflated.

To account for this, the same stochastic device is measured with three different sampling times (shown in Supplementary Figure 9(a)): with sampling times of $\Delta\tau = 3.1\mu s$, $\Delta\tau = 15.5\mu s$, and $\Delta\tau = 77.5\mu s$. Supplementary Figure 9(b) shows the number of counts of middle states (left axis), and the calculated distribution factor (right axis) for each of these sampling times. As expected, the shortest sampling time gives a much larger count of the middle states compared to the slowest sampling time, but the distribution factor is observed to be rather invariant as both the edge and middle states are similarly well-sampled. This proves that it is valid to compare the distribution factor measurements for devices across different dwell time, and sampling time, regimes.

To further validate this quantity, and to see if other correlations might arise, the distribution factor is plotted against other properties of a stochastic MTJ. This includes plotting the distribution factor against the average resistance of the stochastic fluctuation (Supplementary Figure 9(c)), against the magnitude of the stochastic fluctuations (Supplementary Figure 9(d)), and finally, against the bias field where the stochastic fluctuations occur (Supplementary Figure 9(e)). As can be seen in each of these cases, there is no clear correlation between the distribution factor and any other property of the stochastic MTJ other than its unique dependence on the dwell time of the device.

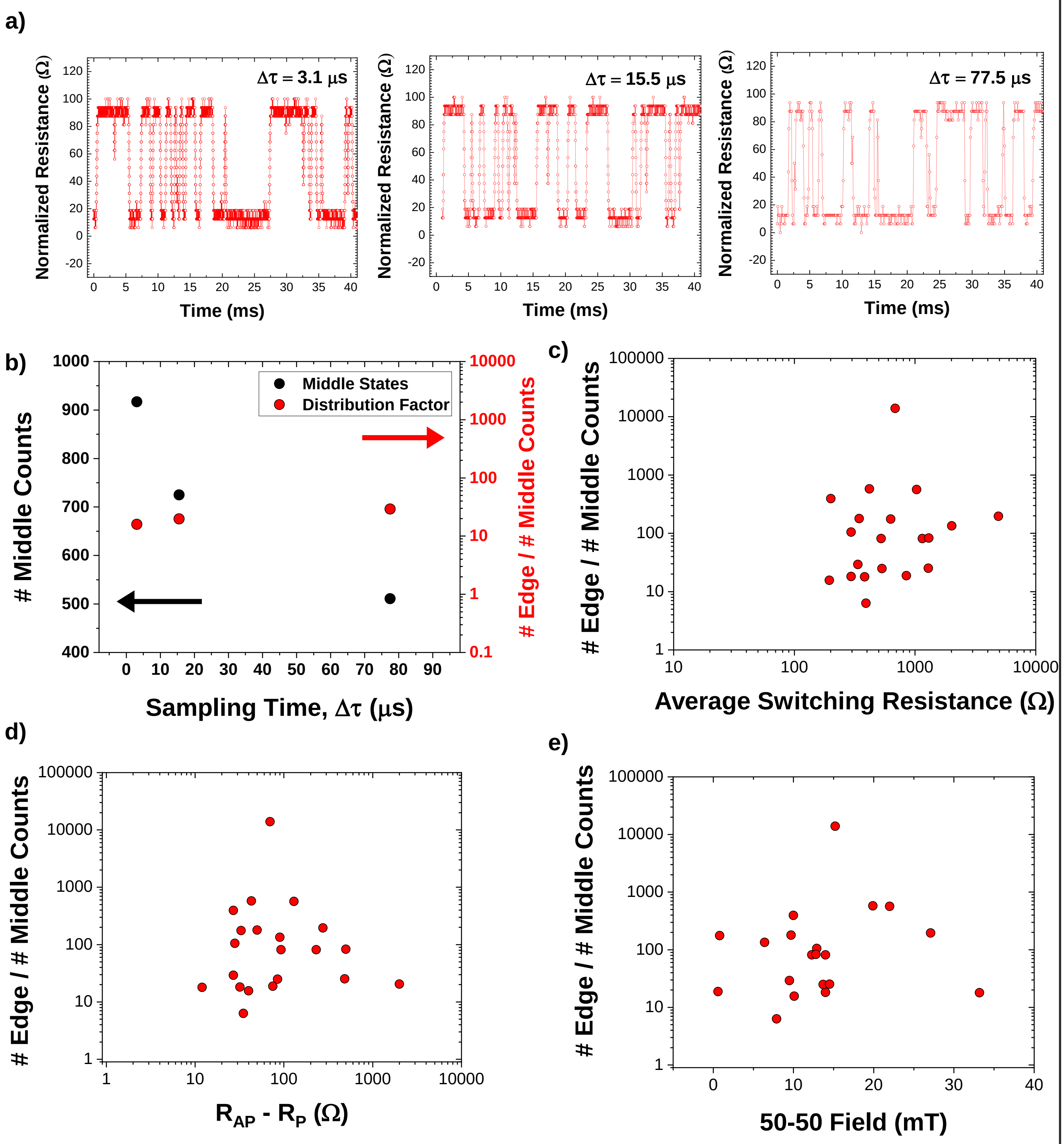

Supplementary Figure 9: Supporting information for the 'Distribution Factor'. a) A series of plots displaying the time-series data taken at the 50-50 point for the same MTJ, but with an increasing sampling time, $\Delta\tau$, between data points. B) Plot showing how the increasing sampling time does lead to a decreasing number of 'middle count' states (left axis), but the 'Distribution Factor' is relatively unaffected (right axis). Graphs plotting statistics from 23 stochastic devices demonstrating how the 'Distribution Factor' of these devices show no correlation with the c) average resistance, d) the magnitude of resistance fluctuations or e) where the stochastic switching is observed to occur (in terms of the externally applied magnetic field). The only correlation observed for this quantity is with the device's dwell time, as presented in the main text.

Supplementary Note 9 – Quantifying the p-bit plateau & its impact on an example "Full Adder" p-circuit

This section details the efforts to develop a framework by which to systematically quantify the level of plateau seen in a p-bit's averaged output, and to then use this method to better analyze how key characteristics of an MTJ can impact the output of the p-bit it is part of.

Supplementary Figure 10(a) shows the averaged output of a p-bit made with an 80%-TMR MTJ (as shown in Figure 4(b) of the main text). The region of interest is defined as the middle 90%-interval of the output, namely the region between V_{OUT} = 0.18V and V_{OUT} = 1.62V. This region of interest is used to define the "ideal" gradient, corresponding to the gradient of the output that might be expected of an ideal p-bit that is devoid of plateaus and is consistently tunable. A plateau is defined as any point within this region of interest where the instantaneous gradient is less than 50% of the value of the "ideal" gradient. The inset dashed lines in Supplementary Figure 10(a) show both the ideal gradient and the gradient below which a point is considered part of a plateau for the 80%-TMR p-bit, while the shaded region shows the part of the output that is highlighted as a plateau by this method. Similarly, the regions identified as plateaus in the 15%-TMR p-bit and the 300%-TMR p-bit outputs are shown in Supplementary Figure 10(b) and 10(c), respectively.

It is observed that the larger the TMR of the device, the wider the range of V_{IN} that corresponds to this middle 90%-interval of V_{OUT} and consequently, the smaller the gradient threshold is to be defined as a plateau. This makes comparing the actual value of the gradients (in the region of interest) across devices of different TMR problematic.

However, and as explained in the main text, one can see that for a fixed distribution of resistance states, the larger the TMR of the MTJ, the wider the plateau region is in the output. To quantify this effect, a "Percentage Plateau" quantity is defined, describing how much of the middle 90%-interval of the output is within the plateau/shaded region.

Supplementary Figure 10(d) shows this quantity as calculated for the 3 devices here. It should be noted that the 15%-TMR device only has an identified 'plateau' at the very edge of the region of interest, where the averaged V_{OUT} begins to saturate as it approaches the output-high state.

In contrast, the output for the 80%-TMR device shows 48% of the central region of interest under a plateau, while the 300%-TMR device shows a plateau region that spans 67% of the central input voltage range.

These wide plateau regions are undesirable for a p-bit, resulting in deviations from the desired tunability of the stochastic output, and a slower time-to-solution for p-circuits (as demonstrated in Kaiser et al. (2022)[5]).

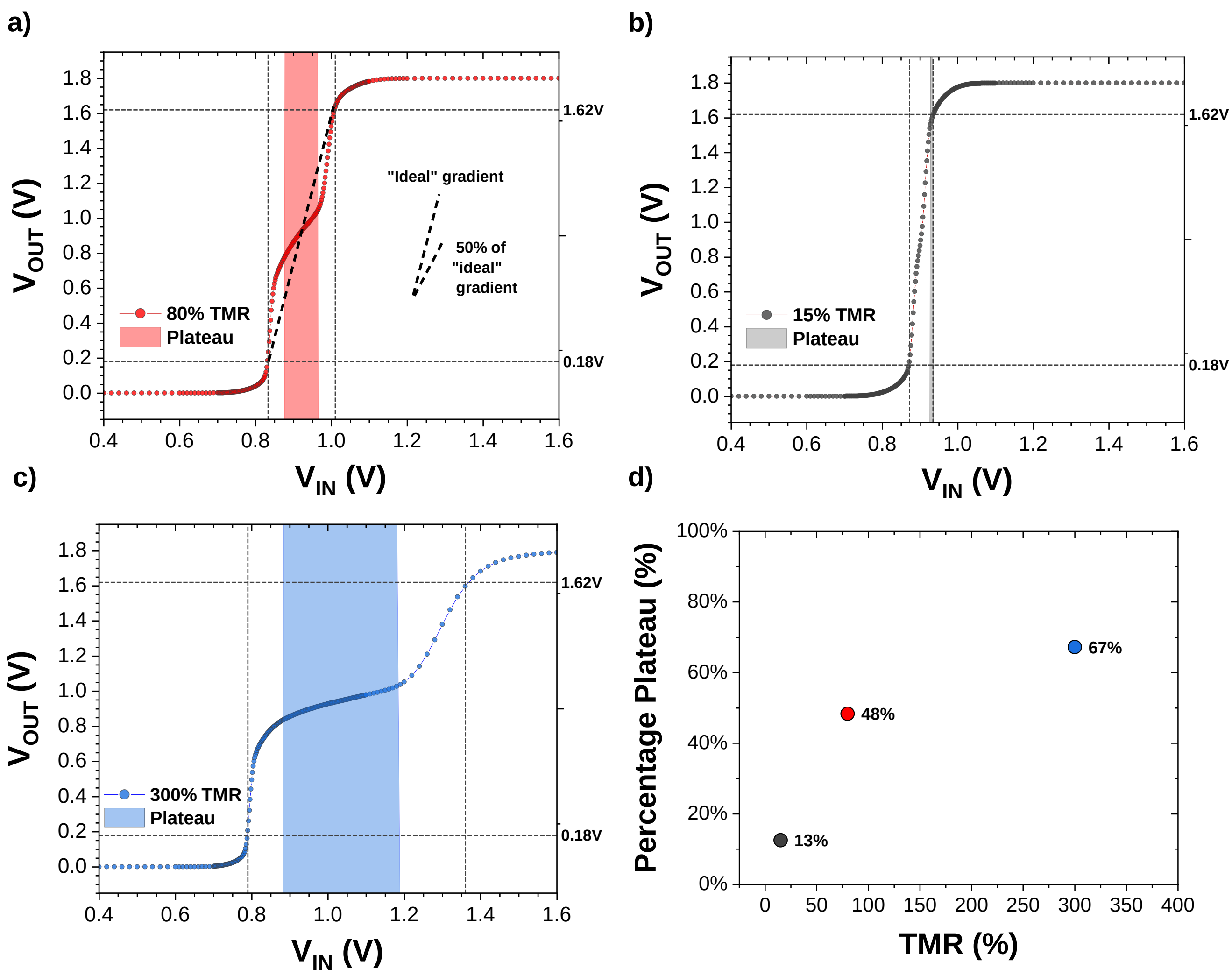

Supplementary Figure 10: Quantifying the p-bit plateau for the impact of TMR on a p-bit's output. a) Averaged p-bit output made with an 80% TMR MTJ, showing how the "ideal" gradient is calculated (over the middle 90%-interval of the output) and how the plateau is defined (as points where the gradient is less than 50% of the "ideal" gradient). The p-bit outputs using b) the 15% TMR device and c) the 300% TMR device are shown, along with the regions identified as a plateau by this method (shaded). d) Graph summarizing how the "Percentage Plateau" (defined as how much of the middle 90%-interval of the output is in the plateau/shaded region) changes with the TMR of the MTJ used in the p-bit. It is observed that the larger the TMR, the more of a plateau is present in the output.

This analysis is repeated for the p-bit outputs shown in Figure 5 of the main text, with the aim of seeing how an MTJ's distribution of states can impact the overall output of the p-bit.

Supplementary Figure 11(a) shows the averaged outputs of two p-bits made with MTJs that are scaled to the same TMR of ~80% but are of different dwell times, τ = 29μs (shown in red) and τ = 27ms (shown in orange). As described in the main text, it is found that the faster device has a distribution of states that is more uniform and less bimodal than that of the slower device, which translates to a "smoother" sigmoidal p-bit output that is less impacted by the plateau problem described previously.

To quantify this, the same method as before is used: the central 90%-interval of V_{OUT} is defined as the region of interest (between V_{OUT} = 0.18V and V_{OUT} = 1.62V) and is the region in which the "ideal" gradient is defined. As before, any point which has an instantaneous gradient that is less than 50% of this "ideal" gradient is defined as part of a plateau region.

Using this method, the plateau regions are identified in the averaged outputs of both p-bits, with the results shown in Supplementary Figure 11(b) (for the τ = 29μs device) and Supplementary Figure 11(c) (for the τ = 27ms device).

As both MTJs are scaled to the same TMR, and use the same transistor and inverter components, the input voltage, V_{IN}, values which define this central region of interest are the same for both devices. This makes quantitively comparing the gradients a valid approach to seeing how the distribution of states can impact a p-bit's output.

As shown in the table (Supplementary Figure 11(d)), the slower device (with a distribution that has an Edge States/Middle States ratio of ~ 81) has a wider plateau region with 58.8% of the central V_{IN} range belonging to a plateau region. This contrasts with the τ = 29μs device (which has an Edge States/Middle States ratio of ~ 6) for which only 18.8% of the central operating region is defined as a plateau.

Aside from just the width of the plateau regions, one can also see a difference in the average gradients within these plateau regions: the slower device is much shallower, with an average gradient of 2.40 within the plateau region, compared to the faster device's output, which has an average gradient of 4.03 within the plateau region.

This translates to the slower device not only having a wider plateau region within its output, but a region in which the stochastic output is much less tunable than that of the faster device. This is especially problematic when concatenating p-bits to form p-circuits.

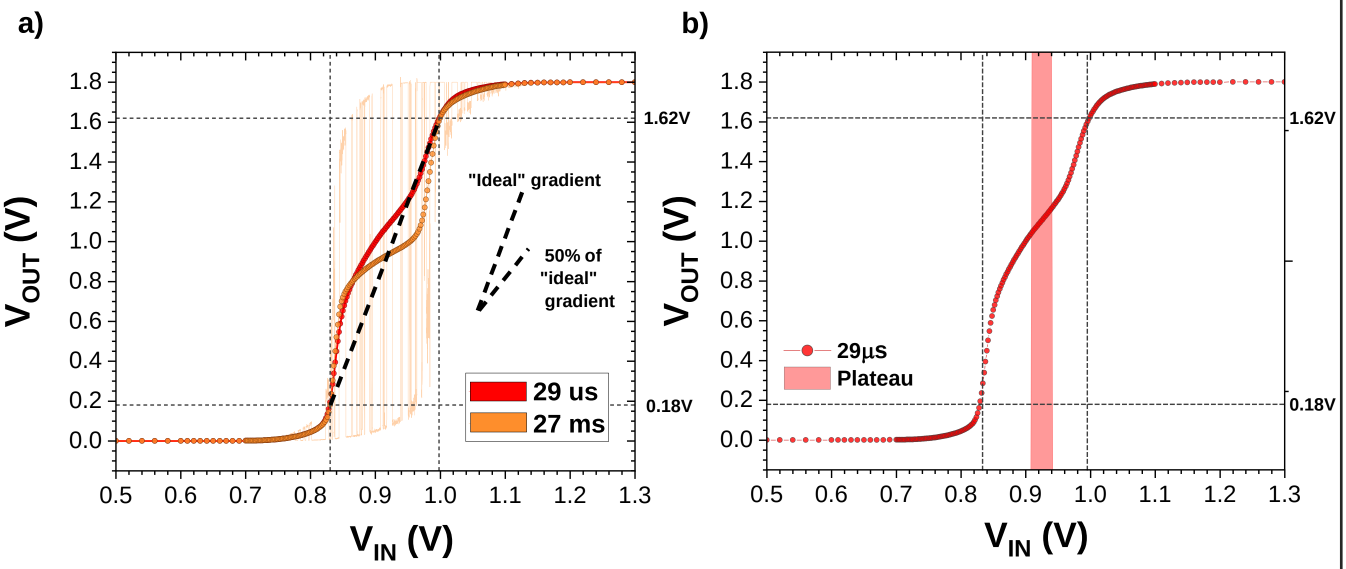

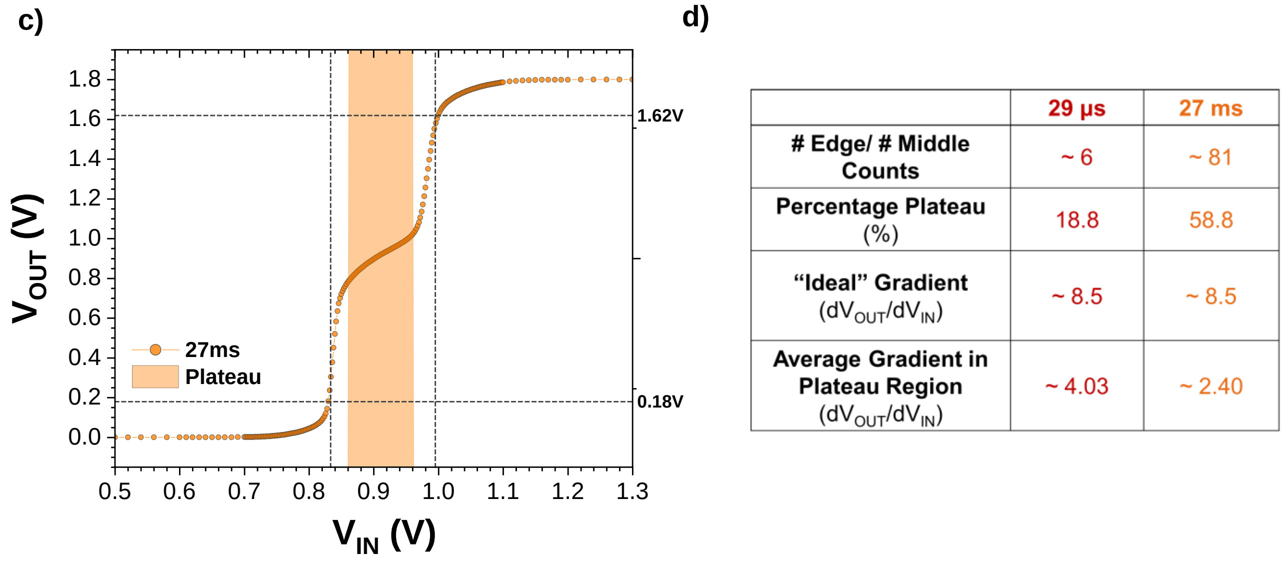

d)

	29 µs	27 ms
# Edge/ # Middle Counts	~ 6	~ 81
Percentage Plateau (%)	18.8	58.8
"Ideal" Gradient (dV_{OUT}/dV_{IN})	~ 8.5	~ 8.5
Average Gradient in Plateau Region (dV_{OUT}/dV_{IN})	~ 4.03	~ 2.40

Supplementary Figure 11: Quantifying the p-bit plateau for the impact of distribution on a p-bit's output. a) The averaged output for two p-bits made with MTJs of different dwell times (and distributions), showing how the "ideal" gradient is defined. A plateau is defined as any point that has a gradient less than 50% of the "ideal" gradient. The individual averaged outputs for the p-bits made with the b) 29µs device and the slower c) 27ms device are shown, along with the shaded regions corresponding to the points identified as plateaus in the corresponding p-bit's output. d) Table comparing the key parameters for the two devices, showing how (for two devices of the same TMR) the distribution can impact the width and severity of the plateau seen in the output.

One such example of a p-circuit that uses non-ideal MTJs as the core of the constituent p-bits is in Kaiser et al. (2022)[5], in which 5 p-bits are interconnected to emulate the operation of a Full Adder circuit. In this work, the performance of this non-ideal p-circuit is directly compared to that of a p-circuit made of 5 "ideal" p-bits, generated using a multiplexer and whose output has no plateaus.

It is shown that the p-circuit made of non-ideal MTJs can learn around the non-ideal behavior and arrive at the same emulated Full Adder truth table (the ground state solution) as that of the ideal p-circuit. However, the time to reach the ground state/solution for the non-ideal p-circuit is approximately twice that of the ideal p-circuit, demonstrating that the plateaus in the p-bit output have a direct impact on the performance of the p-circuit.

This section therefore attempts to quantify the p-bit outputs presented in this paper using the same framework as presented previously, and thus, tie the results obtained with these non-ideal p-bits to that which might be achieved by the p-bits presented in this work.

Supplementary Figure 12(a) shows the time-averaged output of the 5 non-ideal p-bits, made of perpendicular MTJs (p-MTJs) and with dwell times ranging from 10ms – 100ms. Each of the individual outputs shows differing degrees of non-ideal behavior.

For statistical purposes, a linear interpolation is used to populate the points in-between the measured points of the original data (shown in Supplementary Figure 12(b)). This generates averaged outputs with more datapoints within the central region of interest (the middle 90%-interval of the output) that also makes identifying the bounds (in V_{IN}) of this central stochastic region more accurate.

The same method of identifying plateaus (as less than 50% of the "ideal" gradient in this central region) is used, with Supplementary Figure 12(c) showing the results for the "m_3" p-bit output.

The "percentage plateau" (how much of this central stochastic region is within a plateau) is then derived for each of these non-ideal p-bits and is shown in Supplementary Figure 12(d), where the dotted line shows the average value of all 5 data points. This value suggests that, on average, the non-ideal p-bits shown in Supplementary Figure 12(a) have a plateau in ~51% of the central stochastic region.

Considering the results in the paper, this suggests that using MTJs with, on average, ~51% of their stochastic output being a plateau would give a 2x increase in the time-to-solution (in comparison to a p-circuit made of ideal p-bits) for a Full Adder p-circuit.

This demonstrates that a plateau in a p-bit's output correlates to a slowdown in the wider p-circuit's performance with further work required to investigate, on a case-by-case basis, how the width and severity of these plateau regions can impact a p-bit network's performance for different applications.

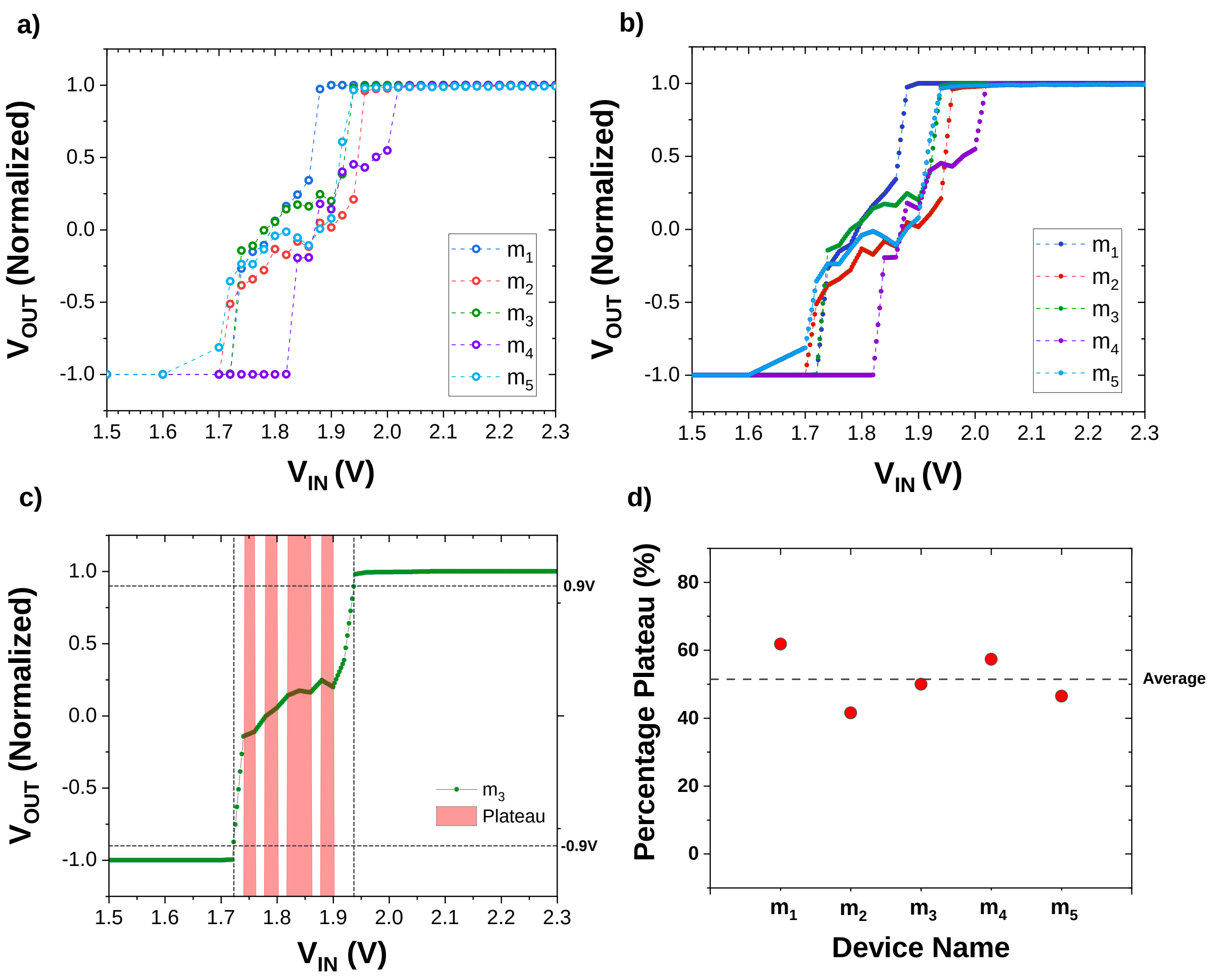

Supplementary Figure 12: Impact of a p-bit plateau on a p-circuit's performance a) Normalized output from the 5 non-ideal p-bits used to emulate a "Full Adder" circuit in Kaiser et al. (2022)[5]. These p-bits are made with perpendicular MTJs (p-MTJs) that have dwell times ranging from 10ms-100ms (reproduced from Kaiser et al. (2022)[5]). b) The interpolated outputs that are used in this work to quantify the level of plateau in each of the non-ideal p-bit's output. c) The output of the p-bit labeled "m_3", showing how the non-ideal regions can be highlighted as a "plateau" by the method described previously. d) The "Percentage Plateau" value (defined as how much of the middle 90%-interval of the output is in the plateau/shaded region) is shown for each of the non-ideal p-bits, along with the average of all 5 devices (dotted line). These 5 devices, with an averaged output that is ~51% plateau, are interconnected to emulate a Full Adder.

Supplementary References

1. Rukhin, A. *et al.* A Statistical Test Suite for Random and Pseudorandom Number Generators for Cryptographic Applications.
2. Ang, S. K. NIST Randomness Testsuit. (2023).
3. Yang, T., Otagiri, M., Kanai, H. & Uehara, Y. Barrier degradation of tunneling magnetoresistance device with MgO barrier and low resistance-area product. *J. Magn. Magn. Mater.* **322**, L53–L56 (2010).
4. Zhao, H. *et al.* Low writing energy and sub nanosecond spin torque transfer switching of in-plane magnetic tunnel junction for spin torque transfer random access memory. *J. Appl. Phys.* **109**, 07C720 (2011).
5. Kaiser, J. *et al.* Hardware-Aware *In Situ* Learning Based on Stochastic Magnetic Tunnel Junctions. *Phys. Rev. Appl.* **17**, 014016 (2022).
